# Mobile EEG for the study of cognitive-motor interference during swimming?

**DOI:** 10.3389/fnhum.2024.1466853

**Published:** 2024-08-29

**Authors:** Melanie Klapprott, Stefan Debener

**Affiliations:** ^1^Neuropsychology Lab, Department of Psychology, University of Oldenburg, Oldenburg, Germany; ^2^Cluster of Excellence Hearing4All, University of Oldenburg, Oldenburg, Germany; ^3^Fraunhofer Institute of Digital Media Technology, Oldenburg Branch for Hearing, Oldenburg, Germany

**Keywords:** mobile EEG, participant mobility, device mobility, cognitive-motor interference, swimming, ecological validity

## Abstract

Research on brain function in natural environments has become a new interest in cognitive science. In this study, we aim to advance mobile electroencephalography (EEG) participant and device mobility. We investigated the feasibility of measuring human brain activity using mobile EEG during a full-body motion task as swimming, by the example of cognitive-motor interference (CMI). Eleven participants were given an auditory oddball task while sitting and swimming, with mobile EEG recording ongoing brain activity. Measures of interest were event-related potentials (ERPs) elicited by experimental stimuli. While the auditory N100 was measured to verify signal quality, the P300 to task-relevant stimuli served as a marker of CMI effects. Analyzes were first performed within subjects, while binomial tests assessed the proportion of significant effects. Event-related changes in the time-frequency domain around turns during swimming were analyzed in an exploratory fashion. The successful recording of the N100 in all conditions shows that the setup was functional throughout the experiment. Regarding CMI, we did not find reliable changes in P300 amplitude in different motor settings in all subjects. However, we found plausible modulations in the alpha/mu and beta bands before and after turns. This study shows that it is generally feasible to measure mobile EEG in the time and time-frequency domain in an aquatic environment while subjects are freely moving. We see promising potential in the use of mobile EEG in extreme settings, advancing toward the application of mobile EEG in more real-life situations.

## 1 Introduction

Over the last decades, research on the human brain has mainly been conducted in highly controlled and stationary laboratory contexts. Based on this work, fundamental knowledge about the anatomical and functional organization of the brain has been generated (Ladouce et al., 2017; Matusz et al., 2019). However, these artificial circumstances do not reflect the situations of everyday life, in which humans move and interact with their surroundings (Chang et al., 2022). It has been recognized in neurocognitive research that motor actions contribute to cognitive processes, which are again enclosed in the motor system. This interaction of the motor system and cognitive systems is generally known as motor cognition (Jeannerod, 2006). Technical advances in experimental setups have enabled cognitive science in motion, termed as mobile brain/body imaging (MoBI; Gramann et al., 2011; Jungnickel et al., 2019). The development of mobile EEG devices has been key to advancing MoBI (Gramann et al., 2011; Debener et al., 2012; De Vos et al., 2014; Bleichner and Debener, 2017), allowing brain research to take place in more naturalistic settings outside of the lab and while participants are moving, elevating the level of ecological validity of cognitive research (Ladouce et al., 2017).

Mobile EEG studies still vary greatly in terms of participant and device mobility, as well as system specifications (Bateson et al., 2017). For instance, an EEG system where all required devices are wireless and head-mounted or carried by the participant, and they can move freely, is called mobile EEG (Debener et al., 2012). The same label can be given to an EEG system, where the participant walks on a treadmill while the EEG cap is connected via cables to a stationary amplifier (Gramann et al., 2011). While this system has advantages in system specifications as the number of available channels or the sampling rate, the mobility of the participant and the devices is limited. In order to advance toward higher degrees of mobility, while maintaining current levels of temporal and spatial resolution, signal quality, and recording duration of state-of-the-art systems, there is a need to explore the usage and the feasibility of mobile EEG systems in more natural settings (Bleichner and Debener, 2017; Stangl et al., 2023).

To validate the signal quality in such motion settings, one or more quality metrics are required. Examples are the presence of event-related potentials (ERPs) after stimulus presentation (Debener et al., 2012) or the pre-stimulus noise on a single-trial basis (De Vos et al., 2014). A popular use case for out-of-the-lab EEG experiments including motion and eliciting ERPs is the study of cognitive-motor interference (CMI). CMI consists in the decrement of the performance in a cognitive and/or a motor task when performed simultaneously as compared to the single-task case (Al-Yahya et al., 2011; Plummer et al., 2013; Leone et al., 2017). This phenomenon has already been described extensively in various settings, such as walking (Debener et al., 2012; Reiser et al., 2021), cycling (Zink et al., 2016; Scanlon et al., 2019), and even skateboarding (Robles et al., 2021), and slacklining (Papin et al., 2024). Common findings include a poorer performance in both the cognitive and motor task, as well as smaller amplitudes of the P300 ERP component, measured with mobile EEG (Gramann et al., 2011; Debener et al., 2012; Lau et al., 2012; Jain et al., 2013; Enders et al., 2016; Zink et al., 2016; Scanlon et al., 2019; Reiser et al., 2021; Robles et al., 2021; Papin et al., 2024). The P300 occurs ~300–600 ms after a stimulus presentation and is known to vary in morphology with stimulus properties, a person's workload and attentional resources, as well as internal states and processes (Polich and Kok, 1995; Polich, 2007; Luck, 2014). A reduced amplitude of the P300 during cognitive-motor dual-tasking (CM-DT) thus implies that an individual's cognitive resources available for the cognitive task are reduced as compared to the single-task setting, due to the additional allocation of resources to the execution of the motor task (see e.g., Leone et al., 2017; Reiser et al., 2021).

This pilot study examines the feasibility of mobile EEG to measure CMI during swimming, thereby (a) extending the literature on EEG in extreme settings and CMI with a new type of motor task , and (b) advancing mobile EEG technology toward being applied in more out-of-the-lab settings. Participants performed an auditory oddball paradigm while sitting (single-task condition) and swimming front crawl (dual-task condition). To our knowledge, this is the first study to record brain activity in an aquatic environment, while participants are freely moving. So far, electrophysiological measurements in water have either been related to animal research, such as EEG recordings in sea animals (Yu et al., 2021; Kendall-Bar et al., 2022), and rats (Günther et al., 2022), or to feasibility tests of measuring ECG during swimming and diving (Stauffer et al., 2018; Sun et al., 2022). There is one publication of a pilot study measuring human EEG underwater, however, the authors used a stationary setup without participant mobility (Schneider et al., 2014).

Based on the current state of CMI research, we expected (a) an enhanced P300 amplitude, measured at channel Pz, after target as compared to standard stimuli in both motor conditions (sitting and swimming), (b) a reduced P300 amplitude after target stimuli in the swimming condition as compared to sitting, and (c) a later P300 peak latency after target stimuli in the swimming condition as compared to sitting. In order to evaluate the interpretability of the data, we also investigated the occurrence of the N100, measured at channel Fz, before testing the experimental hypotheses. The N100 can be seen as an index of stimulus detection (Luck, 2014). We further explored whether there are meaningful changes in the alpha / mu and beta frequency bands (8–13 and 15–30 Hz, respectively) at central channels during swimming, as they are generally associated with movement preparation and coordination (Gross et al., 2002; Palva and Palva, 2007; Maksimenko et al., 2018). As turns in swimming are motion sequences disrupting the ongoing rhythmic movements of the athletes, they require an increased mental effort and coordinative preparation as compared to plain swimming. Therefore, we examined oscillatory activity around turns, normalized to a sitting baseline, in order to investigate if power in the respective frequency bands varies with the mental preparation for turns during swimming.

## 2 Materials and methods

### 2.1 Preregistration

The hypotheses, methods and analysis plans of this pilot study were preregistered prior to analyzing the data. The preregistration was submitted on December 14^th^, 2022, and can be found on the Open Science Framework (https://osf.io/qt4mr).

### 2.2 Participants

For this study, data of *N* = 11 right-handed, healthy participants (five female, six male) were recorded in September and October 2022. The sample size was limited by a short period of time in which we could access the university pool of the University of Oldenburg without any public visitors. The participants reported to have no medical or psychiatric conditions, and normal or corrected to normal eyesight. Most importantly, the participants were required to have several years of experience as swimmers. Their age ranged from 16 to 54 (*M* = 32.18, *SD* = 12.05). Participants were recruited via personal contacts and provided their written informed consent prior to participation. They were reimbursed with 10€/h.

The experiment was conducted according to the Declaration of Helsinki and with approval of the ethics committee of the University of Oldenburg (permit-number: Drs/EK2022/040-01).

### 2.3 Materials

Stimuli were presented via the mobile version of the experimental software Presentation (Neurobehavioral Systems, Berkeley, CA, USA, *Version 23.0 10.27.21*), which ran on an Android smartphone (Samsung Galaxy S21 5G, Android version 12, Modelnb SM-G991B/DS), and transmitted via waterproof in-earphones (IPX8 Waterproof in-Ear Earphones, AGBTEK).

EEG data were measured by 28 sintered Ag/AgCl passive electrodes (at the 10–20 sites F3, F4, C3, C4, P3, P4, O1, O2, F7, F8, T7, T8, P7, P8, Fz, Cz, Pz, POz, FC1, FC2, CP1, CP2, FC5, FC6, CP5, CP6, TP9, and TP10). The reference and ground electrodes for the EEG system were placed at FCz and AFz, respectively. The electrodes were inserted into an elastic cap designed without a chin strap, manufactured by Easycap (Easy-cap GmbH, Hersching, Germany), which was more suitable for the given experimental conditions than standard EEG caps with chin straps. Impedances were kept below 20 kΩ, using *Easycap* electrolyte gel (Abralyt HiCl, Easy-cap GmbH, Hersching, Germany). The mobile amplifier (SmartingPro; mBrainTrain, Beograd, Serbia) was tightly attached to the back of the cap and positioned underneath electrodes O1 and O2.

EEG data were recorded by the same smartphone which was also used for stimulus presentation, using the SmartingPro App (mBrainTrain, Beograd, Serbia, *Version 2.2*), with a sampling rate of 250 Hz. Head movements were captured with the Inertial Measurement Units (IMUs), consisting of 3D accelerometers, gyroscopes and quaternions integrated in the mobile amplifier. EEG data, head movement data, and experimental events were synchronized using Lab Streaming Layer (LSL, Kothe et al., 2024) and an internally developed app (Receiva, *Version 1.0.00.9*; Blum et al., 2021). Data were stored in a single XDF file.

**Figure 1:**
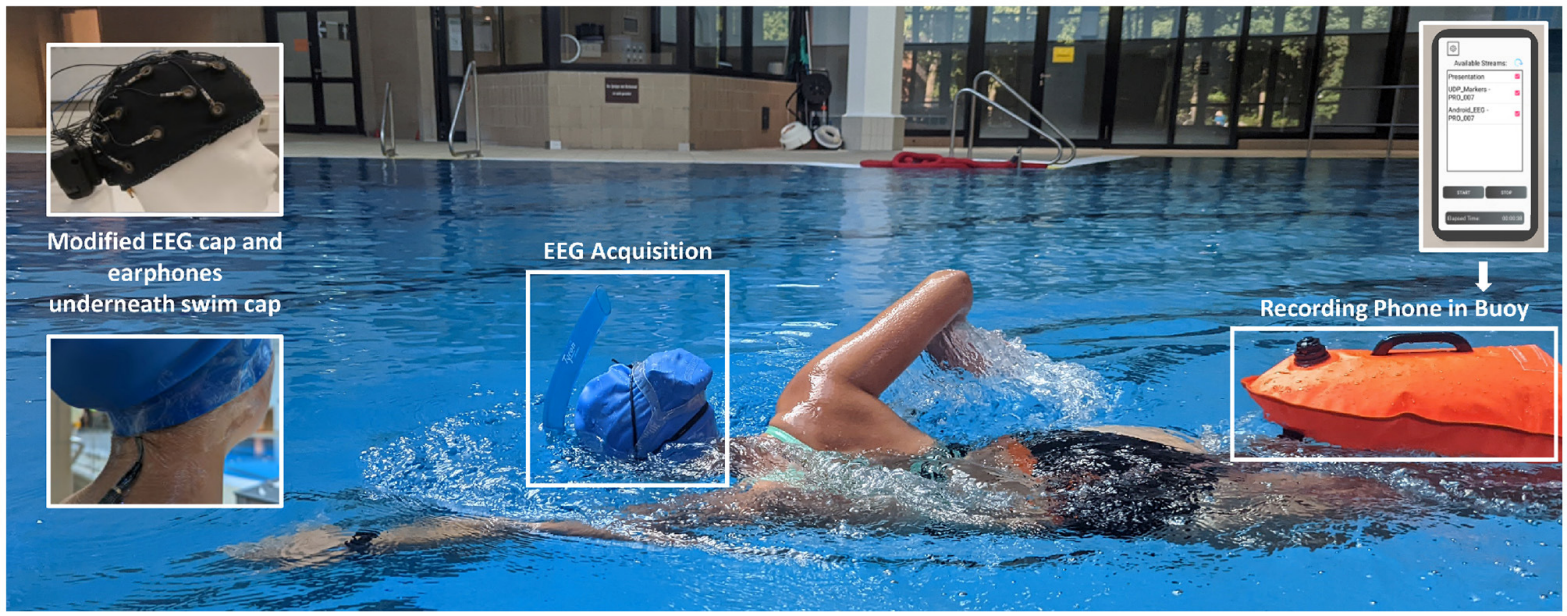
Device setup during the swimming block. Above the swim cap, the subject is wearing her swim goggles and a front snorkel which allows her to breathe without turning her head. Underneath the swim cap lies the EEG cap with the amplifier at the back. Additionally, the subject is wearing waterproof in-ear headphones, which are connected via an elongated audio cable to the smartphone, stored in the buoy. Via the earphones, the experimental stimuli are transmitted from the smartphone to the subject. The smartphone is connected to the amplifier via Bluetooth, in order to synchronize the EEG data, the IMU data from the amplifier as well as the experimental triggers and store them in a single data set. The picture was taken during the piloting process and depicts author MK.

To protect the EEG system from water, a silicone swim cap was pulled over the EEG cap and the amplifier, so that both units were covered by it. Using medical tape, the transition from the swim cap to the participants' skin was sealed. The tape was applied all around the head, as well as to the upper part of the participant's spine, to fixate the audio cable and to prevent water from flowing up from the gaps the cables caused in the seal. The amplifier was additionally protected with a layer of tape around the amplifier box at the back of the EEG cap. For the swimming block, participants were given a front snorkel and a buoy. The front snorkel was worn to allow participants to breathe without turning their heads, keeping the head as stable as possible while swimming and thus reducing head movement artifacts. The buoy served as storage for the smartphone assuring that the smartphone floated above the water so that the Bluetooth connection to the amplifier would not break off. An illustration of the setup during swimming is shown in Figure 1.

### 2.4 Tasks

The cognitive task was a standard auditory oddball paradigm (see e.g., Polich, 2007). Each experimental block consisted of 200 beep-tones of a duration of 70 ms, transmitted via the waterproof in-ear headphones at a participant-controlled, comfortable volume. The interstimulus interval randomly varied between 1 and 3 s. The tones could be standard (a pure tone of 800 Hz) or deviant (1,000 Hz) and were presented in a random order. The number of deviant stimuli varied between 47 and 54 across the blocks. The participants were instructed to count the deviant—target—tones and report the final number to the experimenter at the end of each block.

The motor task demands were manipulated by varying between sitting and swimming as an additional task to the oddball paradigm: during the first ("Pre Swim") and the third ("Post Swim") block, participants were sitting in a chair next to the pool while being given the cognitive task. For the second block ("Swim"), participants were inside the pool and swimming front crawl in addition to the cognitive task. For this block, the auditory oddball was done twice, to have a balanced number of trials between sitting and swimming. This fixed order of blocks was chosen as it was crucial to have a Pre Swim and a Post Swim measurement for each participant, in order to compare signal quality before and after swimming with the EEG device.

### 2.5 Procedure

Data collection took place in the indoor pool of the University of Oldenburg, which was closed to the public during recording times. The water temperature was at ~23°C. After the preparation of the EEG and the swim cap, and an initial resting measurement, the participants performed the first block of the oddball task while being seated, which will be referred to as Pre Swim in the following.

Subsequently, the second block, Swim, started. Participants were given their swim goggles and the front snorkel which were both put over the swim cap. In addition, they were given the buoy, which was attached with a belt around their waist (see Figure 1). Before the oddball during swimming started, the volume of the tones was adjusted to the altered sound conditions underwater. For that, the participants went into the pool and held their face and ears underwater. The experimenter repeatedly played the stimulus sounds until the participants heard them loud enough via the headphones. When the volume was adjusted, a countdown of 5 min started. In that time, the experimenter put the phone into the buoy and the participants could swim for the rest of the countdown to get used to the unfamiliar setup. After the countdown, the oddball task started. Participants swam for ~7 min while doing the oddball task. While the participants were swimming, the experimenter observed them and the technical equipment. After the first half of Swim, a computer voice instructed the participants to swim to the poolside to report the number of target tones they had counted to the experimenter. Then, another 2-min countdown started automatically, during which the participants could either take a break or continue swimming. Following that, the participants again swam for ~7 min while simultaneously working on the oddball task. When the whole block was finished, they were again instructed to swim back to the experimenter to report the counted number of target tones. The experimenter helped the participants out of the pool and brought them back to their chair.

During the last block, in the following referred to as Post Swim, the participants were seated again. As in Pre, the block started with an eye artifact and a resting state EEG measurement, before the participants were given the oddball task. In the end, they were again asked to report the number of target tones they had counted to the experimenter. When the whole experiment was finished, the experimenter took off the swim cap and the EEG cap and showed the participants the way to the showers.

### 2.6 EEG data analysis

**Figure 2:**
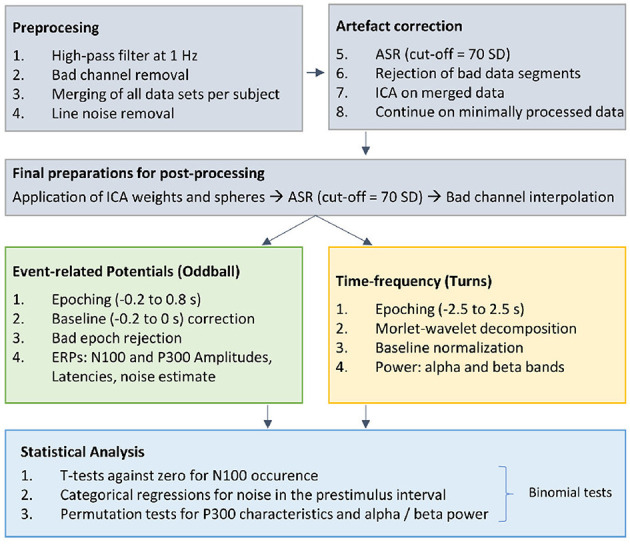
Overview of the EEG preprocessing pipeline. Gray: After basic preprocessing procedures, we applied several artifact correction procedures [artifact subspace reconstruction (ASR; cut-off of 70 *SD*), bad segment rejection based on statistical properties of the data, independent component analysis (ICA)]. The ICA weights and spheres were applied to the “Preprocessed 2” data, which differed from “Preprocessed 1” in terms of filter settings. Green: After re-referencing to the linked mastoid electrodes TP9 and TP10 the data were cut into epochs around the stimuli, corrected for their baseline and bad epochs were rejected to have clean data for the ERP calculation. Yellow: Afer re-referencing to common average, the data were cut into epochs around the turns and normalized to a sitting baseline, to investigate changes in the alpha and beta band. Blue: Depending on the measure, statistical tests were performed.

EEG data were processed using EEGLAB (*Version 2022.0*; Delorme and Makeig, 2004) and custom Matlab scripts (The MathWorks, Inc., Natick, MA, USA, *Version 2022a*). The preprocessing pipeline is illustrated in Figure 2.

#### 2.6.1 Preprocessing

In the first step, the data was investigated in terms of overall completeness. In two subjects (Subject 05 and 08) the recordings of at least one motor condition were missing completely, thus, these two data sets were excluded from further analyzes. In two other participants, EEG recordings of the sitting condition were missing partially (Subject 10: Pre Swim, Subject 11: Post Swim). As the other block of this condition was preserved, respectively, both data sets were included in further analyzes.

After filtering with a zerophase, 1 Hz FIR highpass filter (order 826; EEGLAB function *pop_eegfiltnew()*), bad channels with a standard deviation (*SD*) of their activation exceeding the mean *SD* activation plus 3 standard deviations were identified, visually validated, and temporarily removed. Then, the data of all available blocks for each participant were merged into one file and line noise was removed using zapline plus (Klug and Kloosterman, 2022). The data were cleaned using Artifact Subspace Reconstruction (ASR) with a threshold value of 70 standard deviations. Before the application of an extended infomax independent component analysis (ICA), the data were cut into regularly spaced epochs in steps of 1 s. Of these generated epochs, those with a joint probability of >3 *SD* or with a kurtosis of >3 *SD* were excluded. Using the weights and spheres obtained from the ICA, components capturing eye and muscle artifacts were manually rejected across all experimental blocks per subject. The ICA weights and spheres were applied to minimally processed raw data (0.3 Hz zerophase FIR highpass filter (order 2750; EEGLAB function *pop_eegfiltnew()*), 30 Hz zerophase FIR lowpass filter (order 110; EEGLAB function *pop_eegfiltnew()*). Subsequently, ASR with a threshold value of 70 standard deviations was applied and the temporarily removed channels were interpolated.

For the ERP calculation, the ICA corrected data were then re-referenced to the mastoid channels (TP9 and TP10) and subsequently epoched from –0.2 to 0.8 s around the stimulus onsets and corrected for their baseline (–0.2 to 0 s relative to stimulus onset). Epochs with a joint probability of >5 *SD* and with a kurtosis of >5 *SD* were rejected. In order to explore potential time-frequency changes around turns, we took the ICA corrected data of the swimming block and re-referenced them to the common average. The data were epoched from –2.5 to 2.5 s around turns. The markers for turns were extracted from the data recorded by the IMU channels integrated in the amplifier. An example of how the markers were extracted is shown in the Supplementary material. Epochs with a joint probability of >5 *SD* and with a kurtosis of >5 *SD* were rejected.

#### 2.6.2 ERP component parametrization and analysis

##### 2.6.2.1 N100 amplitude

To validate the functionality of the experimental setup in general, the occurrence of the frontocentral N100 component after standard and target tones was taken as the measure of interest. To define the N100, the minimum voltage for the average ERP of the channel Fz between 100 and 200 ms after the onset of both standard and target stimuli was determined for every participant in every condition. A ± 50 ms time window was put around the peak, determining the individual search window. Then, for every trial, the minimum voltage was searched in this window and a smaller time window of ± 25 ms was placed around the peak of the N100 to parameterize the N100 in the single trials. In addition to the statistical analyzes, the N100 was visually validated by its topography.

##### 2.6.2.2 P300 amplitude and latency

For the parietocentral P300 component, both the amplitude and peak latency were extracted. To investigate the amplitude of the P300, the maximum voltage for the average ERP of the channel Pz between 300 and 600 ms after target stimulus onset was determined for every participant in every condition. A ± 100 ms time window was put around the peak, determining the individual search window. Then, a smaller time window of ± 50 ms was placed around the peak of the P300 in the single trials and the mean amplitude of this time window was taken as the measure for the P300. It should be noted that the parametrization of the N100 and P300 amplitudes deviates from the pre-registered analysis plan. However, given the characteristics of the present data, we decided to adjust the analysis accordingly. The P300 latency was parameterized as the time from stimulus onset until the amplitude peak. In analogy to the N100 investigation, the P300 was visually validated by its topography, in addition to the statistical analysis.

#### 2.6.3 Exploratory: modulation of frequency bands during swimming

It should be noted that this analysis was not included in the original preregistration, but was thought of during the analysis of the planned hypotheses. For exploring whether we can detect meaningful changes in the alpha / mu and beta band during swimming, we re-sampled the data to a sampling frequency of 60 Hz. The downsampling was performed to stay in accordance with previous research in our group, for which EEG data were synchronized with external motion sensors with a lower sampling rate (Jacobsen et al., 2020). We transformed the epoched data around turns into the time-frequency domain by using morlet wavelets. For further calculations, we took five (fronto-) central channels (FC1, FC2, C3, Cz, and C4) as our region of interest (ROI) and averaged across epochs, the ROI, and participants, obtaining a grand average. To eliminate the f/1 pattern, we normalized the averaged, epoched data by a sitting baseline.

### 2.7 Statistical analyses

Statistical analyses on the N100 amplitudes and P300 amplitudes and latencies were performed in RStudio (RStudio PBC, Boston, MA, USA, *Version 2022.12.0*). For the directed hypotheses, all tests were performed within subjects in the first step. The alpha level for significance was set to 0.05. Finally, binomial tests evaluated the proportion of significant effects found within subjects as compared to the chance level in order to control for multiple comparisons. We used the *qbinom()* function to compute the threshold for statistical significance, i.e., the chance level (*St(*α*) = qbinom(1-*α*, n, 1/c)* × *100 / n*), as proposed by Combrisson and Jerbi (2015). Thus, when all subjects were included (*n* = 9), the chance level was at 77%; for the analyzes where one subject was missing (*n* = 8), the chance level was at 75%. For the exploratory analysis, the tests were performed on the grand average across all subjects. To correct for multiple comparisons here, the Bonferroni correction was applied.

#### 2.7.1 Univariate tests for the occurrence of the N100

To validate that the oddball paradigm and the mobile EEG acquisition was successful in all blocks (Pre Swim, Swim, Post Swim), the N100 was taken as a sanity check. Shapiro-Wilk tests assessed normality of the distribution of N100 amplitudes, before performing one-sided *t*-tests or Wilcoxon rank tests (in case of violations of the assumption of normality) against zero (alternative = less). A binomial test compared the proportion of significant effects in the single subjects for all experimental blocks against the chance level.

#### 2.7.2 Permutation tests for P300 properties

##### 2.7.2.1 Main effect of the stimulus type

For testing the main effect of the stimulus type on the ERP, P300 amplitudes were compared between stimulus conditions using permutation tests. The following analyzes were calculated for each block (Pre Swim, Swim, Post Swim) in each subject:

The mean difference between the P300 amplitudes after target and standard stimuli was calculated for the observed data. Then, the labels of the P300 amplitude values were shuffled, and the mean difference was re-calculated. This procedure was repeated 5,000 times to obtain a null distribution of mean differences between amplitudes following target and standard stimuli. As there were more standard than target tones in the experiment, a random subsample of values with a “standard” label, which was equal to the number of observations with a “target” label, was taken for every iteration of the permutations. The observed mean value was compared to the null distribution. Its relative position served as an indicator for the likelihood of the observed mean if it was assumed that the null hypothesis was true. This is equivalent to the *p*-value (Cohen, 2014). This scheme was also applied in the subsequent analyzes, with the respective adjustments. A binomial test compared the proportion of significant effects found in the single subjects and conditions against the chance level.

##### 2.7.2.2 Interaction effect of motor condition and stimulus type

Subsequently, we tested the interaction effect of the motor condition and stimulus type on the ERP amplitudes, hypothesizing a lower P300 amplitude during the swimming condition than in the sitting condition. We re-coded the data, combining the data from the Pre Swim and Post Swim to “Sit,” while applying no changes to the data from Swim. Then, for both motor conditions, the difference between ERP amplitudes in the time range of the P300 component after standard and target tones was calculated. Then, we performed a permutation analysis on these differences in order to investigate the difference between the target-standard differences in “Sit” and “Swim”. Therefore, the same procedure as described above was applied. In order to test the interaction effect of the motor condition and stimulus type on the ERPs' latency, we used the re-coded data with the motor condition labels “Sit” and “Swim.” We applied a permutation analysis to compare the P300 latencies in the cognitive task only condition and the additional motor task condition.

##### 2.7.2.3 Exploration of brain activity during turns

For the investigation of potential changes in the alpha and beta band during turns, the grand average was divided into 10 bins à 500 ms for the alpha and beta frequency bands, respectively. By using permutation tests, each bin was compared to the mean of all bins from the respective frequency band. Thus, first, the actually observed difference was calculated. Then, the labels of the current bin and the rest were randomly shuffled and the difference was calculated again. This procedure was repeated 5,000 times, leading to a null distribution, against which the observed value was compared. The alpha level was adjusted by considering that the tests were performed as being two-sided and by using the Bonferroni correction, leading to an alpha level of α = 0.0025.

## 3 Results

In total, *N* = 2 subjects were excluded from the analyzes due to problems with the recording software. Thus, *N* = 9 participants remained for the statistical analyzes.

### 3.1 Preprocessing

Considering the remaining subjects, on average 1.11 (range: 0–3) channels were marked as bad and temporarily removed from the data. After artifact correction, on average 21 (range: 20–22) independent components remained per participant. At the end of the preprocessing pipeline, on average 122 standard and 47 target epochs (ranges: 117–126; 44–50) remained in the data sets of Pre Swim per participant, which results in a loss of 15.5% of the data. For Swim, on average 244 standard and 75 target epochs (ranges: 221–266; 69–82) were retained, marking a data loss of 20.25%. Finally, on average 129 standard and 48 target epochs (ranges: 123–134; 43–52) remained for Post Swim, indicating that 11.5% of the data were lost due to preprocessing and artifact correction procedures. For the turn epochs, after preprocessing there were 24 (range: 17–30) out of 25.5 (range: 18–31) events per participant left.

### 3.2 Single-trial noise

**Figure 3:**
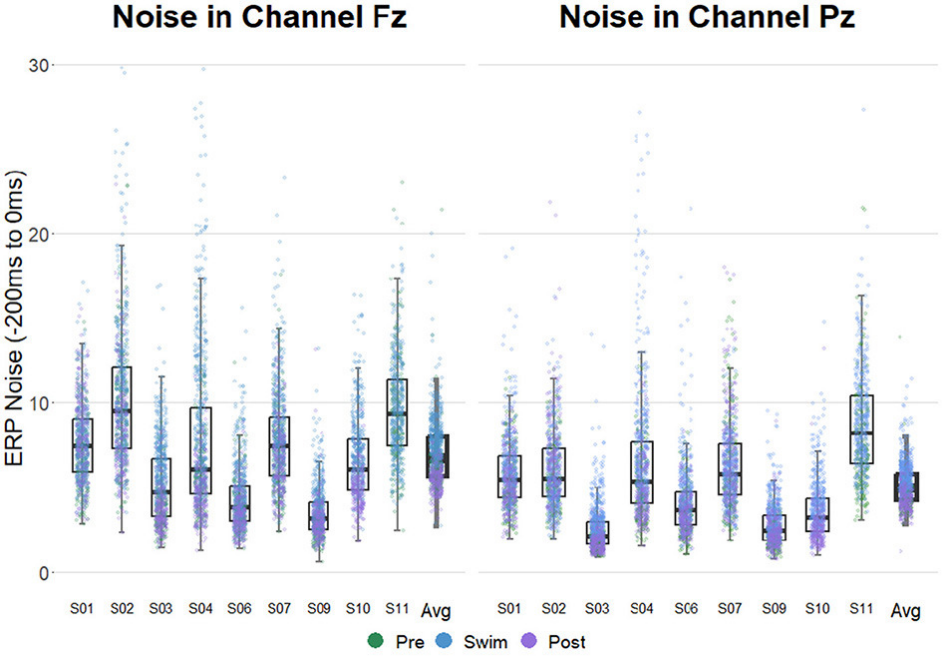
ERP noise levels in electrodes Fz **(left)** and Pz **(right)** in all subjects. The experimental blocks are separated by color. Noise estimates were obtained by computing the standard deviation of the channels of interest in the prestimulus interval on a single trial level. Note that for subject 10 the data of the Pre-block is missing and for subject 11, there is no data available from the Post-block.

Figure 3 shows that noise levels were generally increased in Swim as compared to Pre Swim and Post Swim. Categorical regressions confirmed that in all included participants, the noise level was significantly higher in Swim as compared to Pre Swim (Post Swim for subject 11; *p* < 0.001 for all subjects). A binomial test showed that this proportion does not lie above the chance level (8/8 successes, *p* = 0.1). This result of the binomial test is due to the high chance level threshold resulting from a relatively small n. Despite the effect was reliably significant on the individual level, it did not survive the correction for multiple testing on the group level (*binom.test(8, 8, 0.75, alternative = “greater”)*→*p = 0.1*). There was a significantly higher noise level in Post Swim than in Pre Swim in only two subjects (*p* < 0.05). This proportion does not lie above the chance level (2/7 successes, *p* = 0.99). Note that in this comparison only seven subjects were included as in one subject (subject 10), Pre Swim is missing while in another subject (subject 11) Post Swim is missing.

### 3.3 EEG metrics

**Figure 4:**
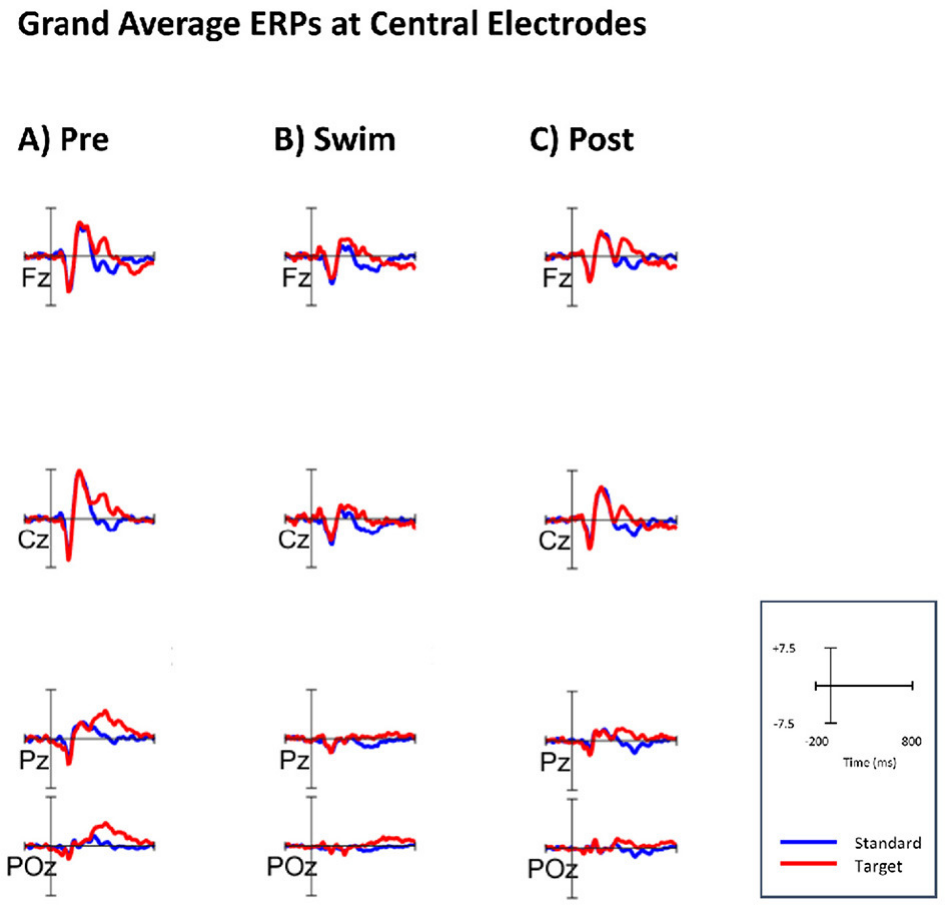
Grand average ERPs measured at central electrodes, referenced to TP9 and TP10. The blue lines indicate the ERPs after standard tones; the red lines indicate the ERPs after target tones. **(A)** ERPs for the Pre condition (sitting). **(B)** ERPs for the Swim condition. **(C)** ERPs for the Post condition (sitting).

**Figure 5:**
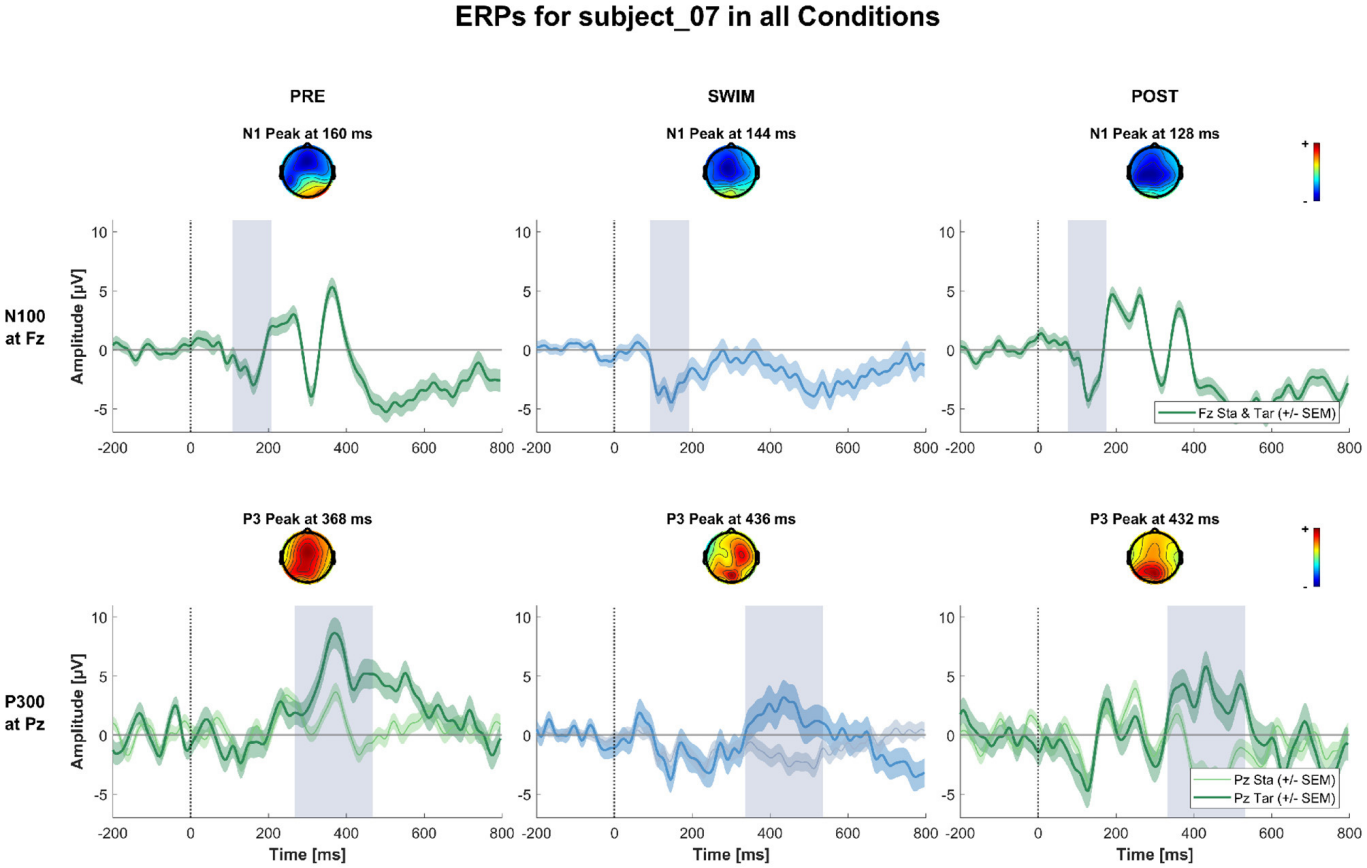
ERPs and topographies in the N100 and P300 windows for all factorial combinations for a subject where all the expected experimental effects could be observed. The upper row shows topographies and channel potential of the N100 measured at Fz for standard and target stimuli combined. The lower row shows topographies and channel potential of the P300 measured at Pz separated by standard and target stimuli. The shaded areas around the channel potentials mark the standard error of the means across single trials. The gray bars around the peak mark the peak of the potential ± or 100 ms, respectively.

**Figure 6:**
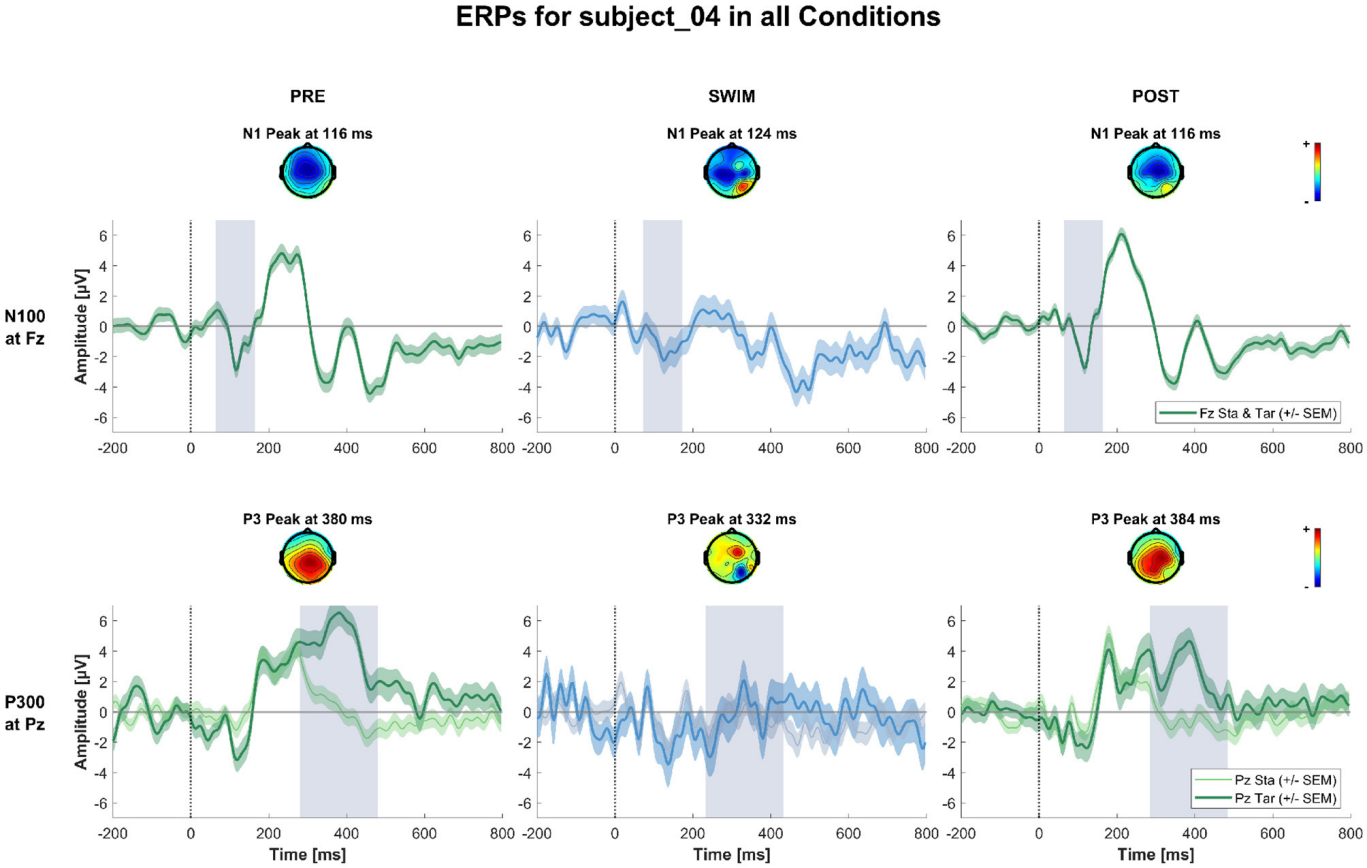
ERPs and topographies in the N100 and P300 windows for all factorial combinations for a subject where the expected experimental effects could be observed during Pre and Post Swim, but not during swimming, due to a decrease in signal quality. The structure is the same as for Figure 5.

**Figure 7:**
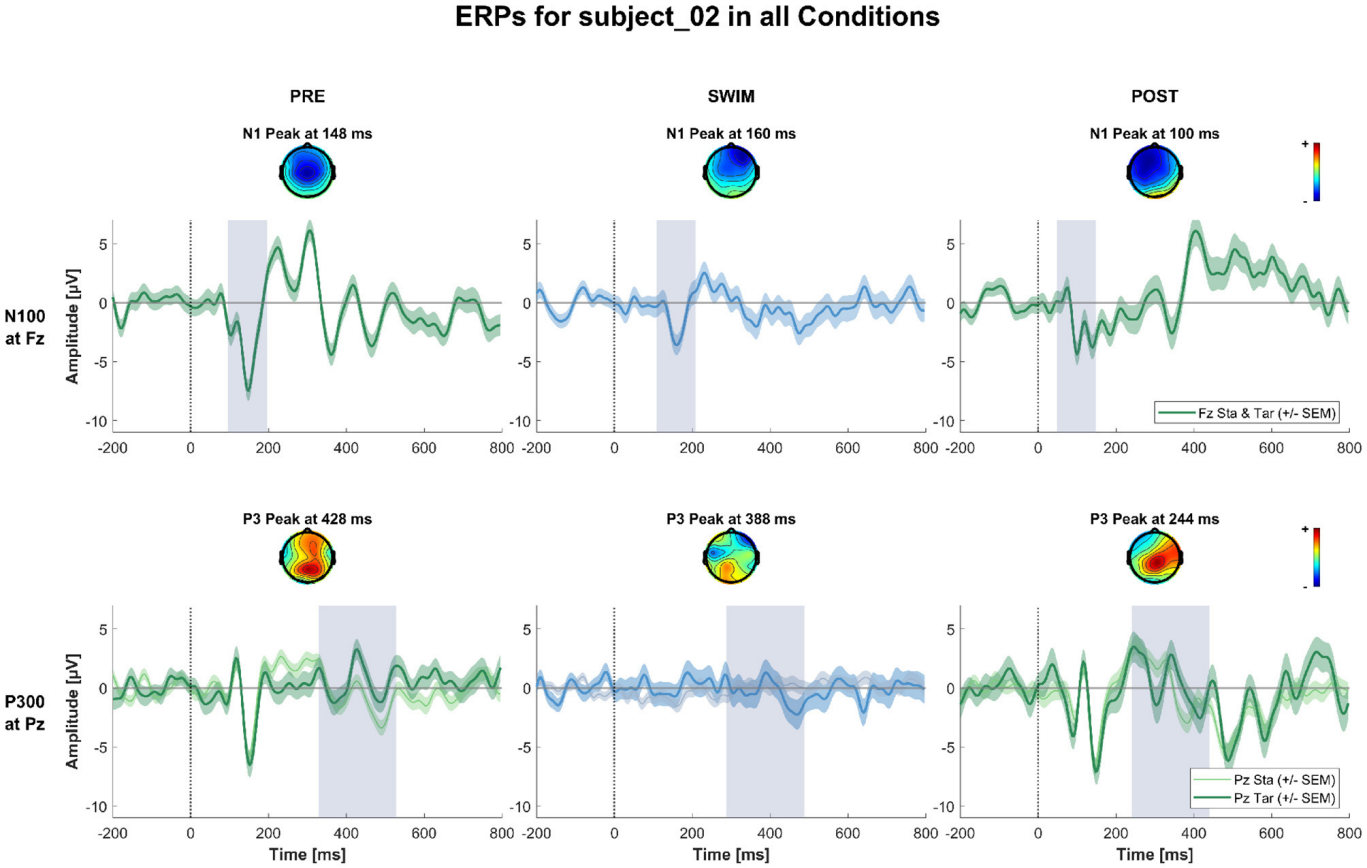
ERPs and topographies in the N100 and P300 windows for all factorial combinations for a subject where the expected experimental effects could not be observed in any of the blocks. The structure is the same as for Figures 5, 6.

**Figure 8:**
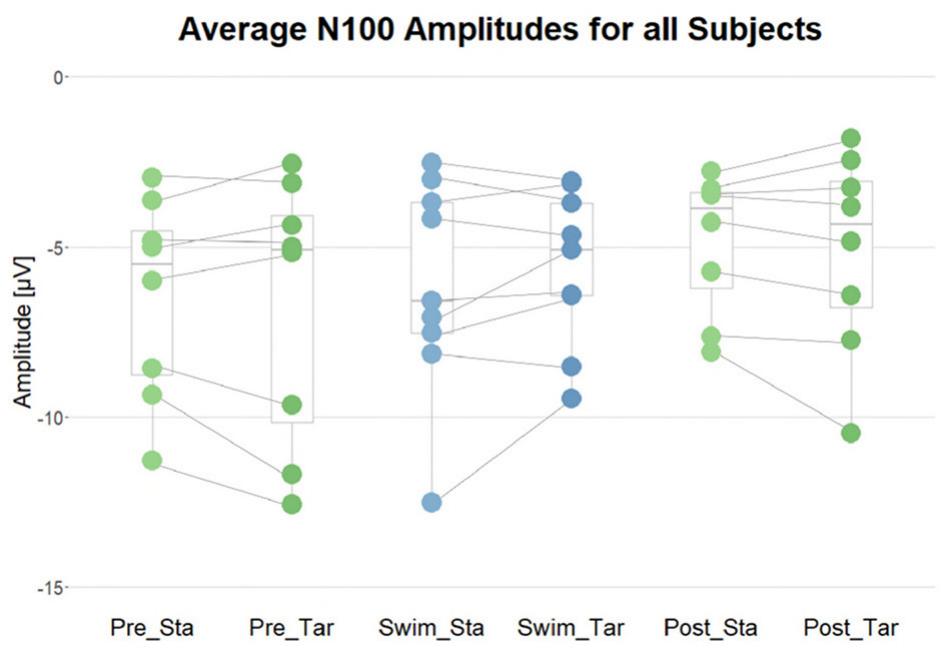
Summary of the average N100 amplitudes after standard (Sta) and target (Tar) stimuli during the experimental blocks. The lines connect the dots belonging to single subjects.

**Figure 9:**
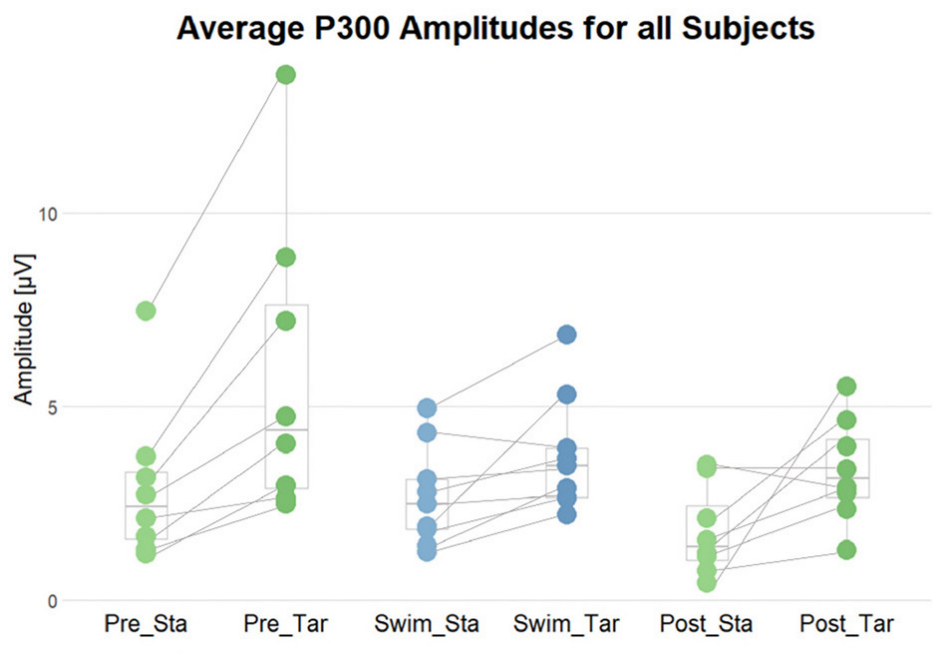
Summary of the average P300 amplitudes after standard (Sta) and target (Tar) stimuli during the experimental blocks. The lines connect the dots belonging to single subjects.

**Figure 10:**
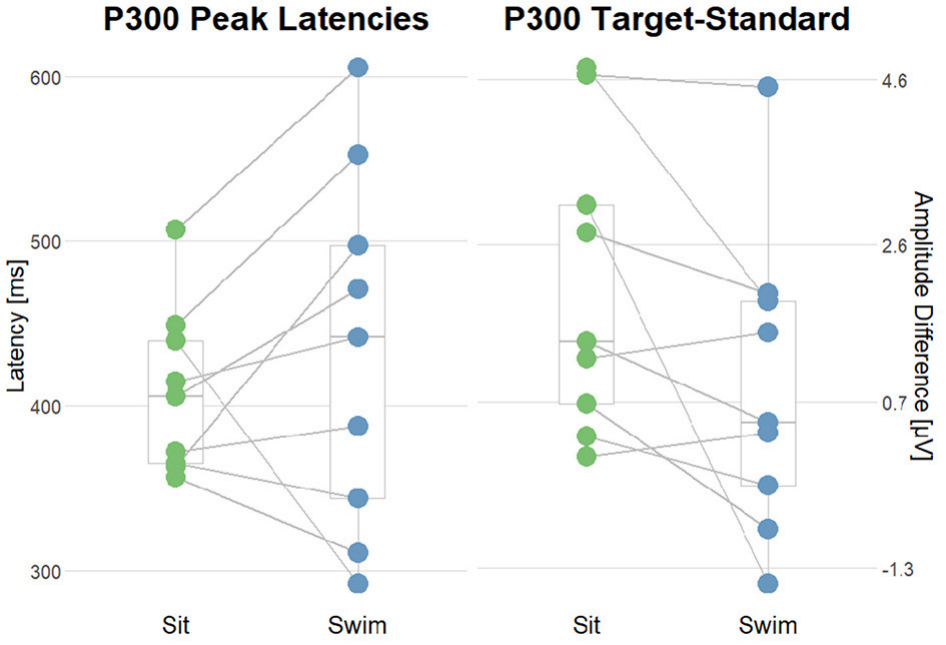
Summary of the interaction effect of the stimulus type and the motor condition on the P300 amplitudes and latencies. The lines connect the dots belonging to single subjects. **(Left)** comparison of P300 Peak latencies after target stimuli in sit and swim. **(Right)** comparison of the amplitude differences (target-standard) in sit and swim. A high score corresponds to a higher P300 amplitude after target stimuli as compared to the amplitude following standard stimuli.

Figure 4 shows the grand-average ERPs at central channels for the standard and target stimuli in all three experimental blocks. Despite the amplitudes are reduced for the swimming condition, one can see typical oddball ERP patterns. In Pre Swim and Post Swim, the ERP waveforms look typical, with only slightly reduced amplitudes in Post Swim. Figures 5–7 show ERPs at channels Fz and Pz, focusing on the N100 and P300, for three examplary subjects, respectively. Figure 5 shows the ERP of a subject, where we found the expected effects in all conditions, Figure 6 shows a subject where the expected experimental effects could be observed during Pre and Post Swim, but not during Swim, due to a decrease in signal quality. Figure 7 depicts the data of a subject who did not show any of the experimental effects at all. The occurrence of the N100 in all subjects and conditions however shows that the experimental setup was functional throughout the experiment. The topographies for the N100 look plausible for all conditions; the topography of the target P300 is only atypical during Swim. Summaries of mean N100 and P300 amplitudes as well as the interaction effects on the P300 amplitude and latency across all subjects are shown in Figures 8–10, respectively. ERP plots including topographies for the subjects not shown here, as well as the grand average and tables summarizing the results can be found in the Supplementary material.

#### 3.3.1 Occurrence of the N100

In order to validate that the setup was functional and that the participants could hear the stimuli well throughout the whole experiment, the occurrence of the N100 ERP component after standard as well as target tones in all blocks served as a criterion. Depending on the distribution of the data within subjects and blocks, one-sided t-tests or Wilcoxon tests against zero were performed. The results show that the N100 occurred in all subjects in all experimental blocks (*p* < 0.05), which is supported by plausible ERP topographies. Binomial tests however show that the proportion of significant findings is not significantly above the chance level (*p* = 0.1 for Pre Swim and Post Swim; *p* = 0.104 for Swim). Despite the effect was reliably significant on the individual level, it did not survive the correction for multiple testing on the group level (*binom.test(9, 9, 0.77, alternative = “greater”)*→*p = 0.104*).

#### 3.3.2 Effects of experimental manipulation on the P300

To test the first hypothesis, permutation analyzes were applied for comparing amplitudes in the time range of the P300 component between ERPs after standard and target tones in all experimental blocks. We hypothesized a higher amplitude in the P300 time range following target stimuli as compared to standard ones. In Pre Swim, we found significant main effects in six of the eight subjects included in the analyzes (*p* < 0.05), which are also represented by the topography. A binomial test however showed that the given proportion does not significantly lie above the chance level (*p* = 0.68). In Swim, we found a significant effect in two of the nine subjects (*p* < 0.05), which a binomial test showed to be below chance level (*p* = 1). In Post Swim, we found a significant effect in 5 of the 8 included subjects (*p* < 0.05), which are also topographically valid. A binomial test however showed that the given proportion does not significantly lie above the chance level (*p* = 0.89).

In order to answer the second research question, whether there was an interaction effect of the motor condition and stimulus type regarding the ERPs' morphology, permutation analyzes were applied on P300 amplitudes and latencies. Firstly, we compared amplitude differences between the standard and target condition in the range of the P300 component between ERPs in the sitting and swimming condition. We hypothesized the difference between target and standard amplitudes to be larger during sitting than during swimming. We found a significant effect in one out of nine subjects. This proportion was not significant (one out of nine; *p* = 1), as assessed by a binomial test. Subsequently, we tested whether there was an interaction effect of the motor condition and stimulus type on the latency of the P300 after target stimuli, hypothesizing an increase in the latencies for P300 peak amplitudes during swimming. For doing so, we performed permutation analyzes on the P300 latencies in Sit and Swim. We found a significant effect in five out of nine subjects. A binomial test evaluated this proportion as not being significantly above the chance level (*p* = 0.97).

#### 3.3.3 Oscillatory activity around turns

**Figure 11:**
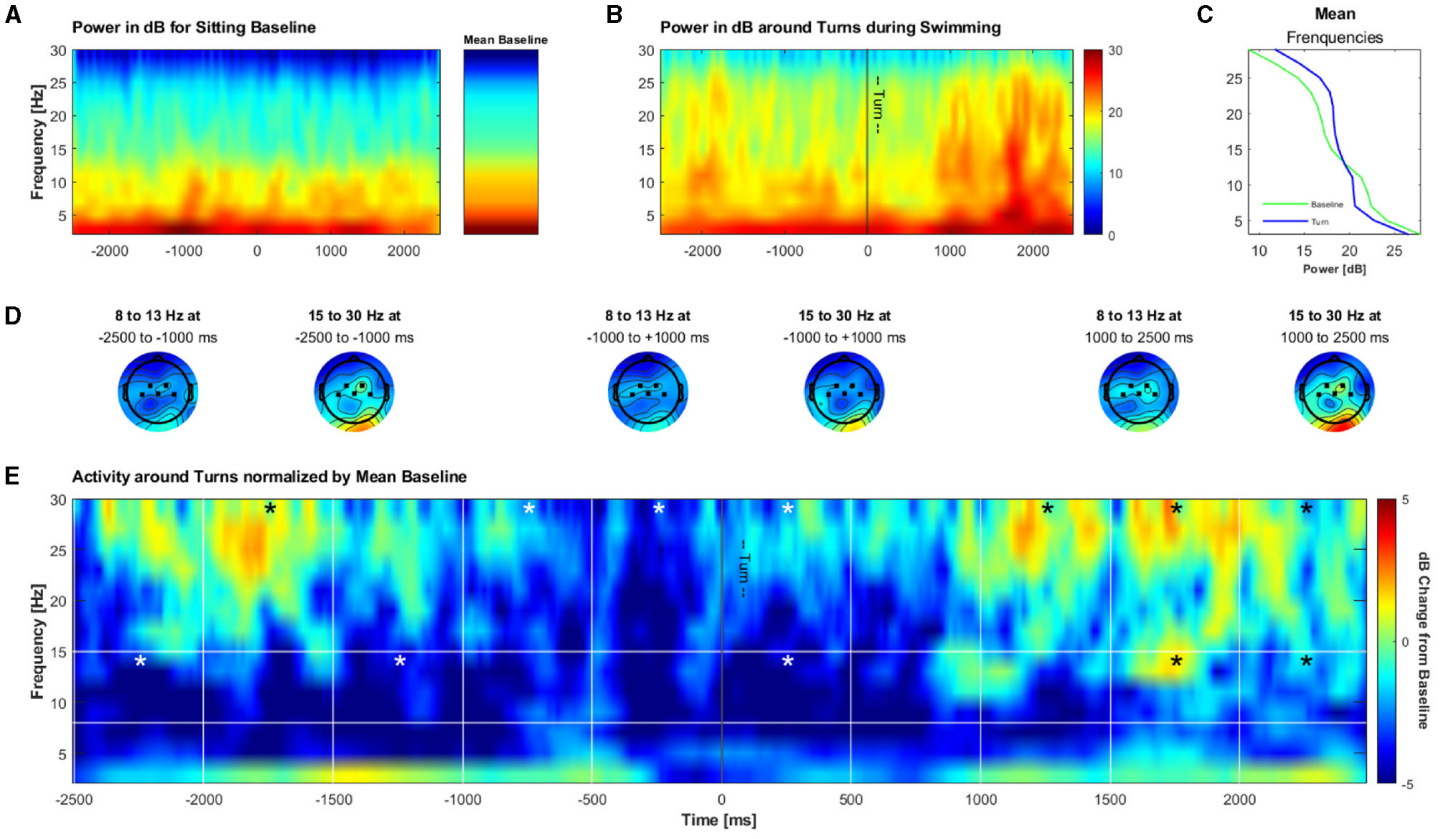
Grand average of the time-frequency solution around turns during swimming. **(A)** Frequency power in dB during an arbitrary 5 s time frame for illustration purposes during an open-eyes resting measurement during sitting and the mean of the power during the whole open-eyes resting measurement. **(B)** Frequency power in dB during turn phases while swimming (turn ± 2.5 s). **(C)** Mean frequency power during the sitting baseline and turn epochs. **(D)** Mean topographies of alpha and beta in 1,500 ms windows during the turn phases. **(E)** Frequency power during turn phases normalized by the mean sitting baseline. Bins marked with a star contain a significantly lower or higher dB change from the baseline as compared to the mean of the whole activation in the respective frequency band.

Lastly, we aimed to explore whether we can measure meaningful data during swimming also in the time-frequency domain. For this we analyzed potential changes in alpha/mu and beta activity from –2.5 to 2.5 s around turns in an undirected, exploratory fashion. Permutation tests of single bins á 500 ms against the whole activation showed a significant decrease in the alpha/mu band from –2,500 to –2,000 ms, and from –1,500 to 1,000 ms before the turn, as well as immediately after the turn (0–500 ms; *p* < 0.0025 in the respective bins). Significant increases in the alpha/mu band as compared to a sitting baseline were found in the bins from 1,500 to 2,500 ms after the turn (*p* < 0.0025 in the respective bins). For the beta band, a significant increase in power was found from –2,000 to –1,500 ms before and from 1,000 to 2,500 ms after the turn (*p* < 0.0025 in the respective bins). A decrease in beta band power was found from –1,000 to 500 ms (*p* < 0.0025 in the respective bins) around the turns. Next to the description of how we obtained these data, the results are illustrated in Figure 11.

## 4 Discussion

This study investigated whether it is possible to acquire meaningful mobile EEG data during swimming, by the example of CMI. Participants performed an auditory oddball task while being seated and while swimming, with their brain activity being constantly measured by mobile EEG. Measures of interest were the ERPs elicited by the experimental stimuli in all conditions as well as modulations of the alpha/mu and beta frequency bands during turns in the swimming pool on an exploratory basis.

### 4.1 Feasibility of measuring mobile EEG during swimming

In order to obtain the data at hand, we had to overcome considerable challenges. First of all, the integrity of the EEG system had to be guaranteed for the whole experiment, especially during swimming. By developing a custom EEG cap without chinstraps, and isolating it with an off-the-shelf swim cap and medical tape, we successfully made the system waterproof. Second, the Bluetooth connection between the mobile amplifier and the phone recording the data had to be stable, just as the presentation of the auditory stimuli of the oddball paradigm. The phone had to be close to the participants and always above the water surface, as (a) Bluetooth cannot transfer data through water, and (b) the audio cable could not be too long in order to be properly fixated at the participants' back. In addition to instructing the participants to not dive after turns, this setup enabled a stable Bluetooth connection and stimulus presentation via the headphones. Lastly, the data quality had to be sufficient to obtain meaningful results from the study. The main issue here were the head movements involved in front crawl swimming. By giving the participants a front snorkel that allowed them to breathe without turning their heads, we avoided greater head motion, which led to more stability and a higher signal quality of the EEG. Another potential problem could have been sweat artifacts, which are known to detoriate the EEG signal. However, we did not observe these artifacts in our data, possibly due to the rather low temperature of the university's indoor pool (23°C).

The subsequent analysis of the data shows that we were successful in overcoming these challenges and that it is indeed feasible to measure meaningful human EEG data during swimming. The analysis also showed that the recorded data profited from common mobile EEG preprocessing procedures. Despite the increased data loss after preprocessing (20.25%) and noise levels in Swim, we found a reliable N100 component of the ERPs in all participants, as well as P300 components in a part of the participants. Even if these effects did not survive the correction for multiple testing due to a too small sample size, we clearly see the ERPs and topographies on the single subject level. Further, it was possible to obtain meaningful data in the time-frequency domain around the turns during swimming. Finally, the maintained quality of the data in Post Swim shows the robustness of our system in an aquatic environment.

Regarding the degrees of mobility postulated by Bateson et al. (2017), it lies at hand that we reached considerably high levels of device mobility (headmounted + smartphone for recording) and participant mobility (free movement except for the head). On the side of the system specifications, we reached a rather moderate level (unshielded, passive gel electrodes; 24-bit resolution; sampling rate of up to 500 Hz; ~5 h battery life), which, however, was sufficient for our purposes. The use of active electrodes for instance would also not have been feasible, as they would be too large to fit properly underneath the swim cap. As a recent study showed that the signal quality of passive electrodes does not significantly differ from the quality of active electrodes (Scanlon et al., 2021), we do not see any disadvantage of our system in this regard. In comparison to the studies considered by Bateson et al. (2017), and other more recent MoBI studies (Mavros et al., 2022; Studnicki et al., 2022; Liu et al., 2024) our setup offers an advanced combination of device and participant mobility. Despite there are setups with advantages in terms of system specification, these cannot compete with the degree of overall mobility our system offers (for an illustration, see Bateson et al., 2017).

### 4.2 Effects of CMI during swimming

In previous studies, it was consistently shown that target tones elicited a larger P300 amplitude than standard ones (Gramann et al., 2011; Debener et al., 2012; Lau et al., 2012; Jain et al., 2013; Enders et al., 2016; Zink et al., 2016; Scanlon et al., 2019; Reiser et al., 2021; Robles et al., 2021; Papin et al., 2024). Further, the authors reported CMI measurable on the brain level, shown in a reduction in P300 amplitudes in dual-task conditions as compared to single-task conditions. In how far CMI is affected by swimming on the brain level, however, remains open, as our results are not clearly in line with the existing literature.

To begin with, even in the sitting condition (especially in Post Swim) not all participants showed the expected main effect of the stimulus type. This could have been due to inter-individual differences and the finding that not all humans show a P300 (Woodman, 2010; Luck, 2014), or intra-individual variations, as cognitive and arousal-related states (Polich and Kok, 1995), which might have an increased influence in a study with a rather small sample size. Another reason could be that the participants did not understand the task. However, this explanation is rather unlikely, as the participants reported a realistic number of target tones they had counted and especially in Post the task should have been clear. A more realistic explanation would be that while still highly motivated in the first block of the experimental paradigm, the participants' engagement in the task decreased over time, contributing to overall lower amplitudes in Post Swim. Further, the decrement of the P300 amplitude may be due to physical exertion. Despite the participants were instructed to swim carefully and relaxed, we cannot exclude that for some participants the swimming task was more exhaustive than for others. Previous research has shown that a high intensity bout of physical exercise can have a negative impact on cognitive performance (e.g., Pontifex et al., 2019, thus, which may also be reflected in ERP amplitudes). Regarding the absence of the oddball effect on the P300 during Swim, it might be that the dual-task was merely too difficult for most participants and that they prioritized the motor task rather than focusing on the cognitive task. This would be in line with the “posture first” strategy (Shumway-Cook et al., 1997), postulating that in CM-DT situations individuals prioritize the motor task over the cognitive task to maintain physical integrity. Thus, this concept might be extended to a “not drowning first” strategy. However, as the experiment did not contain any questionnaires for the participants about the perceived difficulty of the task, we cannot be sure whether this was the case in this study. Nonetheless, we do see an effect in some of the subjects (see also the Supplementary material), so the recording circumstances in single sessions also must have played a role, alongside with inter- and intra-individual variations (Polich and Kok, 1995; Luck, 2014).

Concerning the P300 peak latency, we could not find a clear association of altered motor conditions and the components' latency. In a part of the participants, we found the expected effects, but in the other part, the direction of the effect was reversed. This inconsistency has also been reported in earlier studies (Ladouce et al., 2019; Kao et al., 2020; Papin et al., 2024). It should be kept in mind that in our study, we focused on effects on the subject-level, while the previous studies mentioned above investigated the effects on the group-level. More research, also combining both approaches, would be beneficial to further understand the discrepancies between the findings. However, the reader should also note that in our study, the CMI effects on the P300 amplitude were not reliable; thus drawing conclusions about effects on the P300 latencies should not be made.

### 4.3 Exploratory: oscillatory activity around turns

In addition to the time domain, we investigated the brain activity during swimming in the time-frequency domain. Specifically, we explored whether there are changes in the alpha / mu and beta band as a correlate of cortical preparations for turns, measured at central channels. We indeed found significant deviations over time in both the alpha / mu and beta frequency band, indicating beta desynchronization just before the turn and beta synchronization from 1 s on after the turn, as well as a synchronization in the alpha / mu band ~1 s after the turn. Both frequency bands change in different manners, indicating that it is not likely that the observed patterns are purely due to noise and artifacts.

Movement is associated with desynchronization in the alpha / mu and beta frequency bands (Gross et al., 2002; Maksimenko et al., 2018), thus it is not surprising to see that especially in the alpha / mu band there is less power as compared to the sitting baseline. The decrease in beta just before the turn might reflect the immediate preparation for it. The slight increase in in both frequency bands in the remaining time windows might be explained by a re-synchronization of oscillatory activity, especially after the turn. As, to our knowledge, this is the first examination of brain activity during a complex full body motion, we cannot link these results to any prior research or interpret this finding unambiguously. Thus, more research on brain activity during complex, full body movements is needed in order to properly interpret and support our findings in this regard.

### 4.4 Limitations

Despite the impact of our research for potential future applications of mobile EEG in cognitive and sports neuroscience, there are some limitations to the study that should be noted. First of all, the small sample size indicates that the observed effects have to be interpreted with caution. However, a larger sample size was not possible for this pilot study, as we only had access to the university pool for a few weeks, while the piloting and fine-tuning of the setup took an extensive amount of time during that period. Due to the limited sample size, more detailed analyzes regarding for example demographic characteristics like potential age or gender differences were not feasible. Another limitation is the prototypical setup we designed for the experiment. For instance, there was only one modified EEG cap available, which was used for all participants, so the the electrodes did not sit at the exact same position for all participants, due to their variations in head size. This might have contributed to some irregularities in the data and between single participants.

On the side of the experimental design, we have to mention that the order of the blocks was not randomized or counter-balanced between participants, thus, the study is not immune against potential order effects. As it is known that the P300 amplitude habituates after some repetitions of the same experiment, (Lammers and Badia, 1989) this also might have contributed to the reduced oddball effect in Post Swim. However, the aim was to study the feasibility of acquiring mobile EEG data during swimming, thus, a Pre and a Post measurement was needed for all participants. Last but not least, there was no valid behavioral component included in the study, such as a reliable behavioral performance during the oddball task or questionnaires about the perceived difficulty of the respective conditions. Despite the participants were asked to report the number of counted target tones, it might be that they had guessed at some point which we did not have control over. The reason for this implementation was that a reliable measure such as button presses would not have been possible, especially during the swimming block.

### 4.5 Conclusion and outlook

To conclude, this pilot study not only extends the literature on CMI, but also shows that it is feasible to measure EEG in such a hostile environment as water. So far, investigations regarding swimming were either limited to stationary diving (Schneider et al., 2014), dry conditions (Shi et al., 2017; Mikicin et al., 2020), ECG or other behavioral recordings in humans (Stauffer et al., 2018; Stets et al., 2020), or recordings in animals (Yu et al., 2021; Günther et al., 2022; Kendall-Bar et al., 2022). Future research with more mature setups for measuring mobile EEG during swimming might enable more thorough diagnostics in the swim sport, complementing existing methods, such as general procedures like video taping, or more advances techniques to also measure muscle fatigue, as tensiomyography (Buoite Stella et al., 2024). Another branch of research could investigate more effects of swimming on cognition and brain activity. As there is a growing body of research showing benefits of swimming on cognition and brain health (Abou-Dest et al., 2012; Lin et al., 2020; Faíl et al., 2022), it would be interesting to complement the effects reported on a behavioral level and on the animal brain with data recorded from the human brain. Despite there is still a lot of potential for technological and methodological advancements, we are confident that those can be achieved and enable more mobile EEG research in sports and health science.

## Data Availability

The raw data supporting the conclusions of this article will be made available by the authors, without undue reservation.
