# Supplementary material for: Mobile EEG for the study of cognitive-motor interference during swimming?
Source: Front Hum Neurosci. 2024 Aug 29;18:1466853. doi: 10.3389/fnhum.2024.1466853 (PMC11390454; doi:10.3389/fnhum.2024.1466853)
Supplement: Supplementary file 1 [file Data_Sheet_1.PDF]

Supplementary Material

1 SUPPLEMENTARY TABLES AND FIGURES

Supplementary Figures S1 to S6 show the event related potentials (ERPs) and topographies for the remaining subjects that were not shown in the main text. Supplementary tables S1 S2 and S3 summarise the statistical analyses performed on the Noise estimate and the P300 metrics investigated in this study, respectively.

Supplementary figure S7 shows an example of how the events of turns during swimming were derived. Supplementary figures S8 and S9, as well as supplementary table S4 summarise the permutation tests performed on the time-frequency data around turns.

1.1 Noise Statistics

		Estimate	Std. Error	*t*-value	*p*-value
Subject 01	Intercept (Pre)	6.066	0.123	49.321	<.001 ***
	Swim - Pre	1.052	0.153	6.885	<.001 ***
	Post - Pre	0.577	0.173	3.346	0.001 **
Subject 02	Intercept (Pre)	7.600	0.208	36.594	<.001 ***
	Swim - Pre	0.942	0.256	3.683	<.001 ***
	Post - Pre	0.272	0.293	0.931	0.352
Subject 03	Intercept (Pre)	2.723	0.106	25.739	<.001 ***
	Swim - Pre	2.322	0.130	17.826	<.001 ***
	Post - Pre	-0.108	0.148	-0.728	0.466
Subject 04	Intercept (Pre)	5.316	0.253	21.050	<.001 ***
	Swim - Pre	4.457	0.307	14.531	<.001 ***
	Post - Pre	-0.628	0.352	-1.781	0.075
Subject 06	Intercept (Pre)	3.576	0.104	34.515	<.001 ***
	Swim - Pre	1.319	0.128	10.287	<.001 ***
	Post - Pre	-0.189	0.148	-1.282	0.200
Subject 07	Intercept (Pre)	6.534	0.145	45.018	<.001 ***
	Swim - Pre	0.848	0.177	4.780	<.001 ***
	Post - Pre	0.243	0.204	1.193	0.233
Subject 09	Intercept (Pre)	2.359	0.067	35.318	<.001 ***
	Swim - Pre	1.336	0.082	16.245	<.001 ***
	Post - Pre	0.354	0.094	3.777	<.001 ***
Subject 10	Intercept (Swim)	5.848	0.089	65.640	<.001 ***
	Swim - Post	-2.204	0.153	-14.376	<.001 ***
Subject 11	Intercept (Pre)	8.363	0.158	52.913	<.001 ***
	Swim - Pre	1.195	0.195	6.124	<.001 ***

Table S1. Subject-wise summary of the difference between ERP noise estimates for the single experimental blocks. Noise levels differ significantly between Pre Swim and Swim, while Pre and Post Swim only differ significantly in noise levels for two participants. This underlines the maintenance of signal quality after swimming with our mobile EEG system.

1.2 Subject-wise ERPs

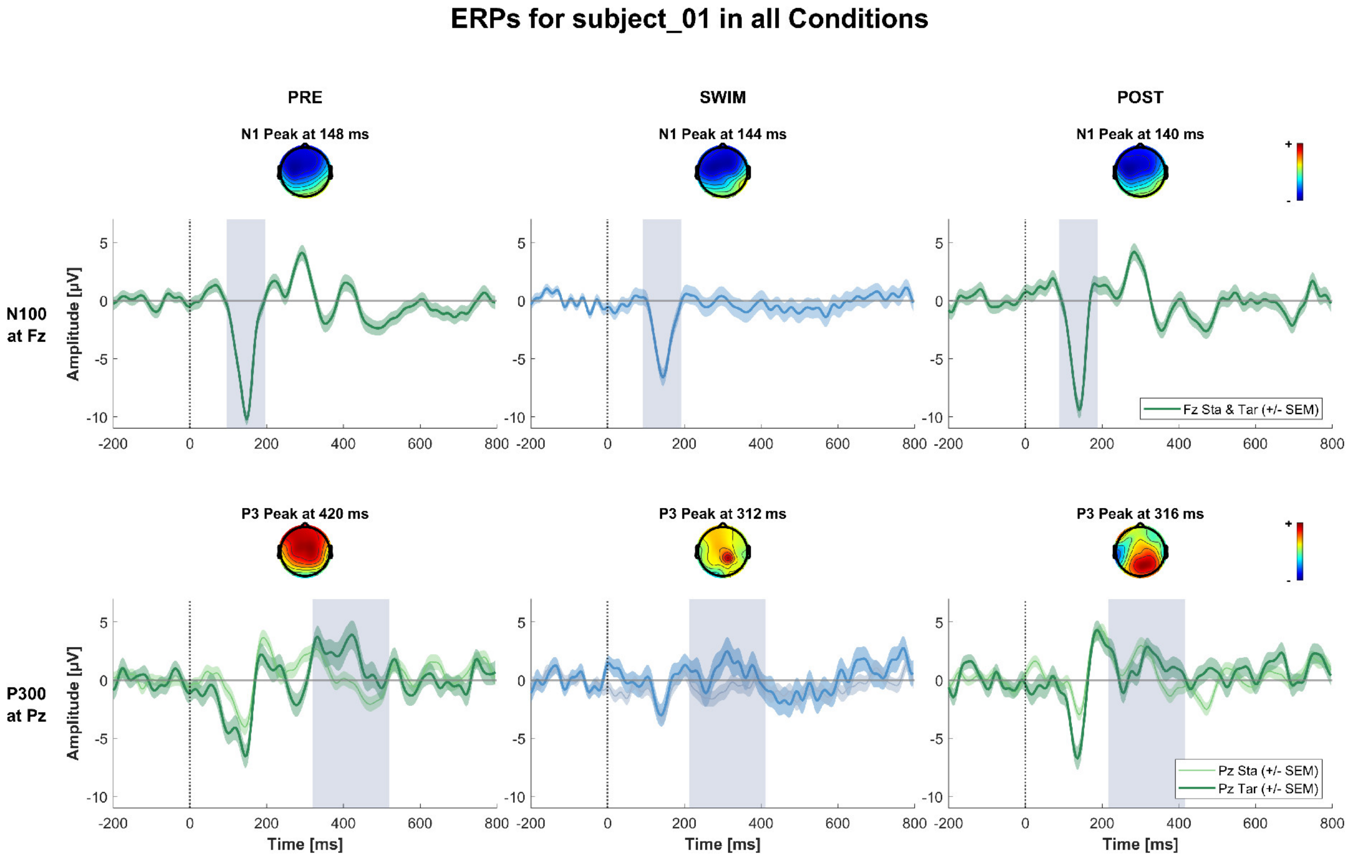

Figure S1. ERPs in every condition for Subject 01. While the N100 is present reliably in every condition, the oddball effect on the P300 amplitude is only present in the Pre Swim block. The interaction effects proposed on the P300 amplitude and latency are not found here.

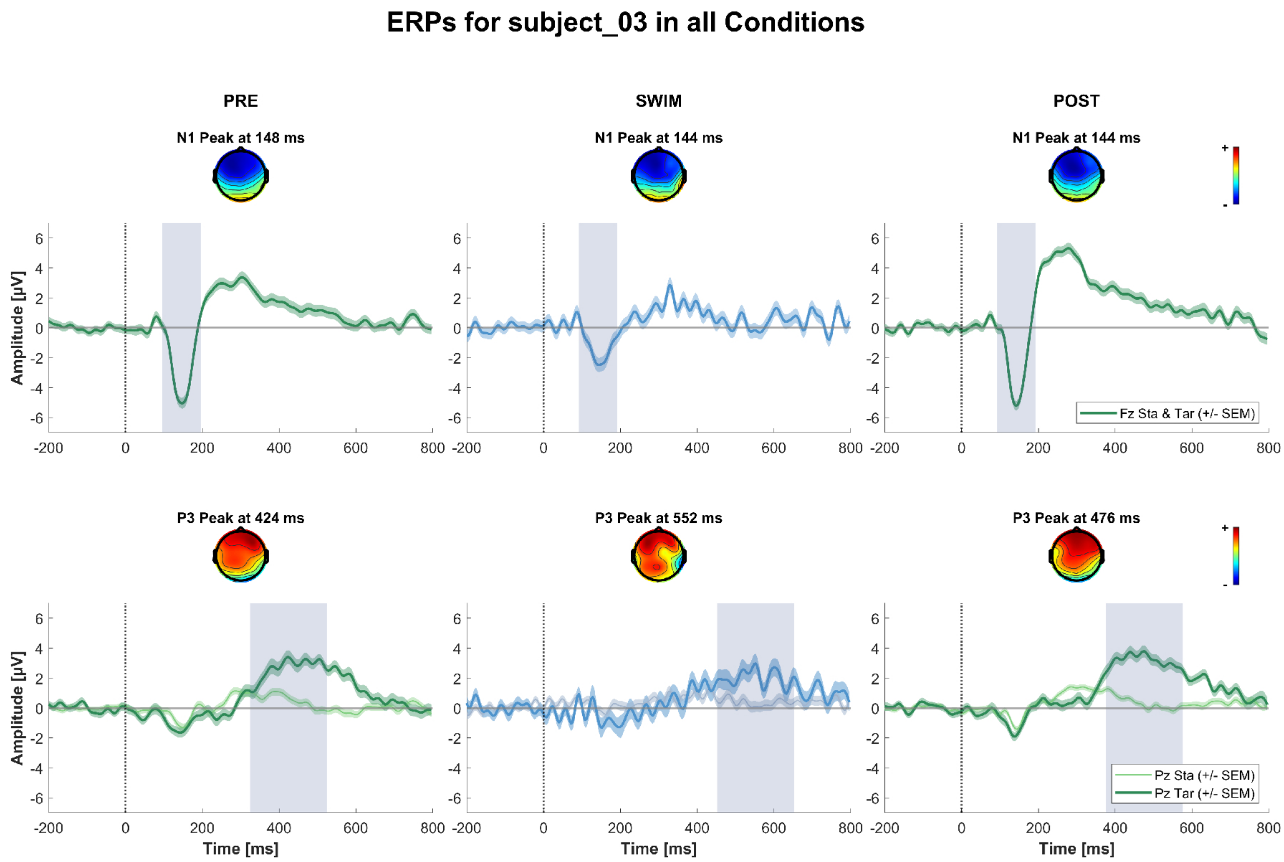

Figure S2. ERPs in every condition for Subject 03. In this subject, the expected N100 and oddball effects on the P300 are present. However, the amplitude difference between standard and target tones does not differ significantly between the sitting and swimming conditions.

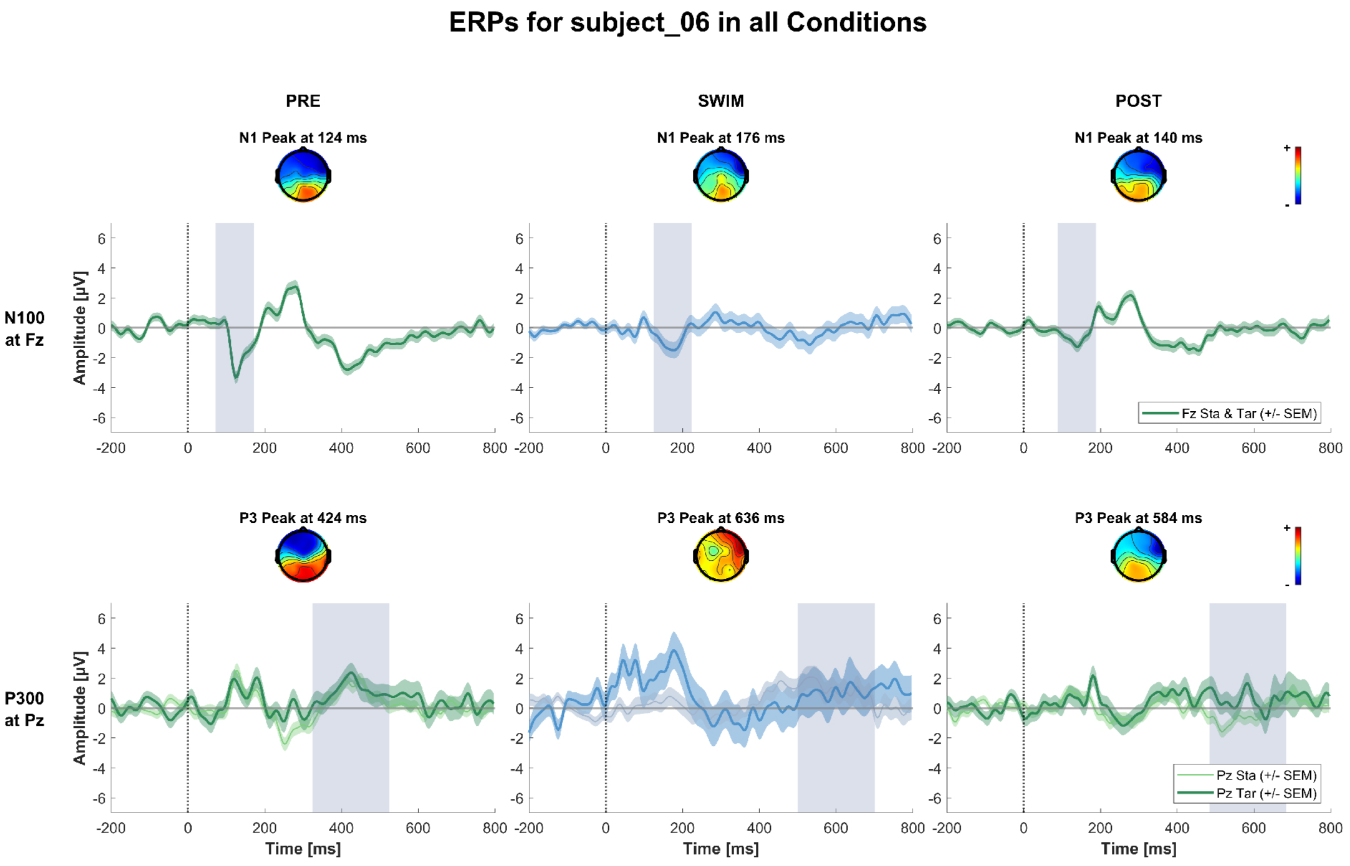

Figure S3. ERPs in every condition for Subject 06. The N100 is present in each experimental block. The expected main and interaction effects on the P300 are not found in any of the blocks.

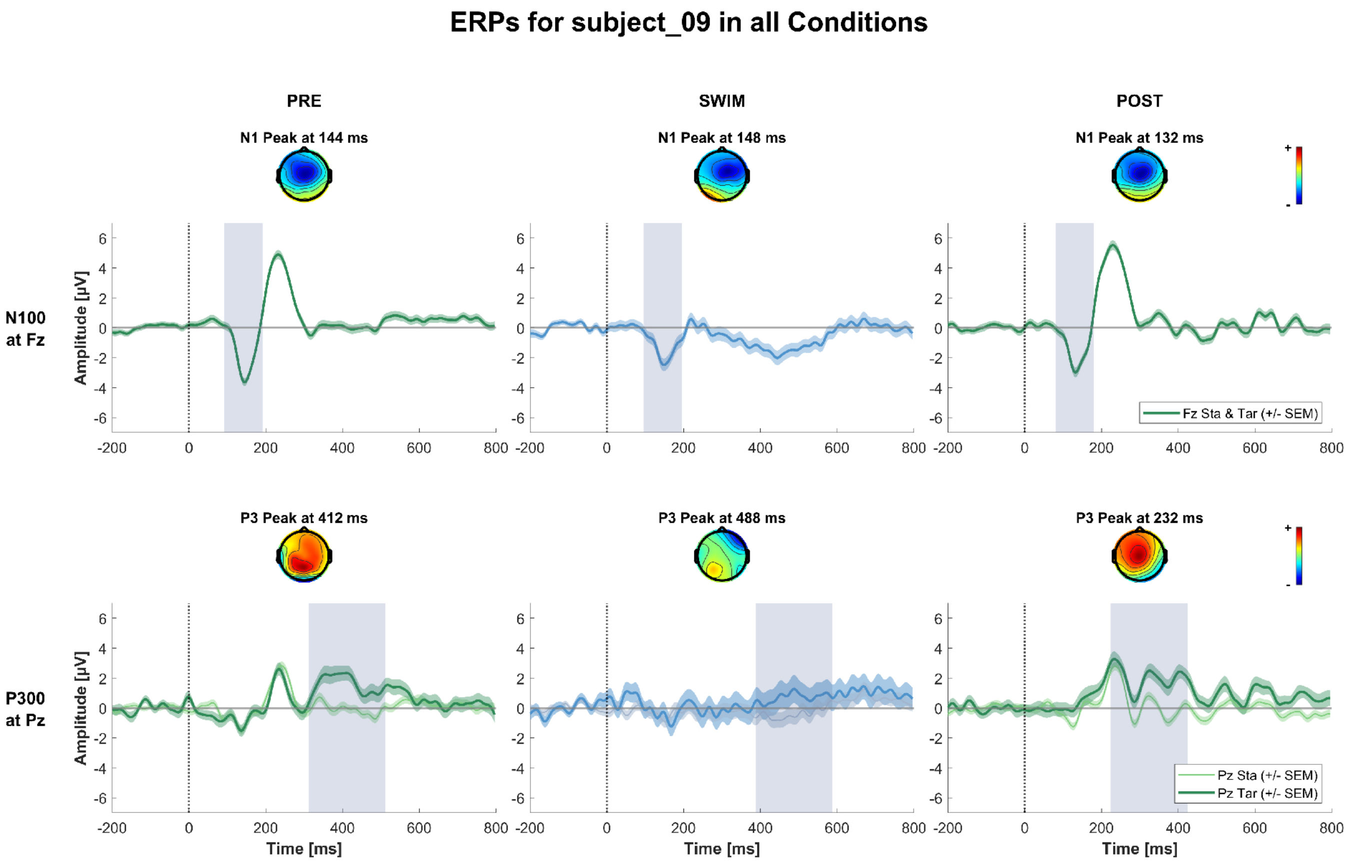

Figure S4. ERPs in every condition for Subject 09. The N100 is present in every block. In the Pre and Post Swim blocks, the oddball effects on the P300 amplitude can be found. In Swim, the difference did not show significance. However, the latency of the P300 during Swim is significantly later than during the sitting blocks.

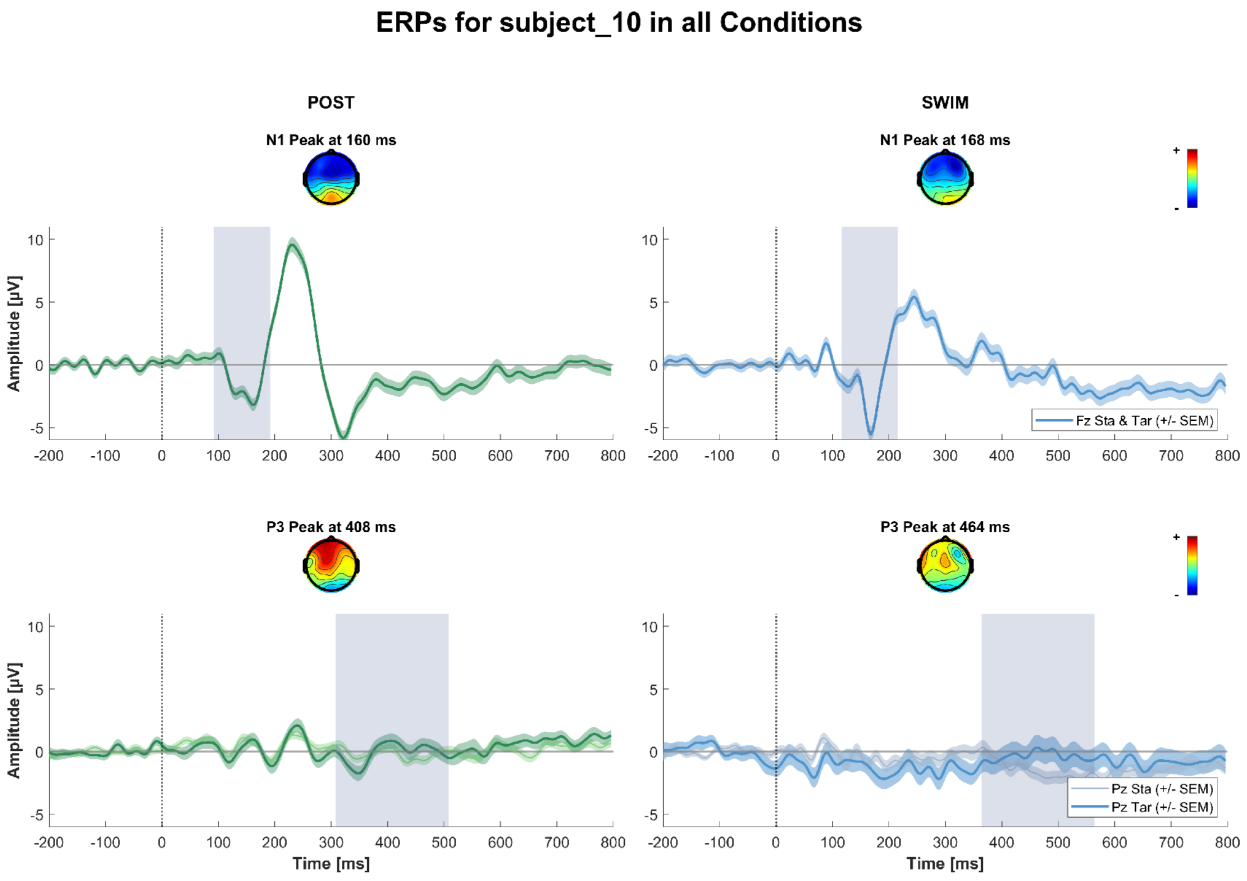

Figure S5. ERPs in every condition for Subject 10. While the N100 is present, there are no effects on the P300.

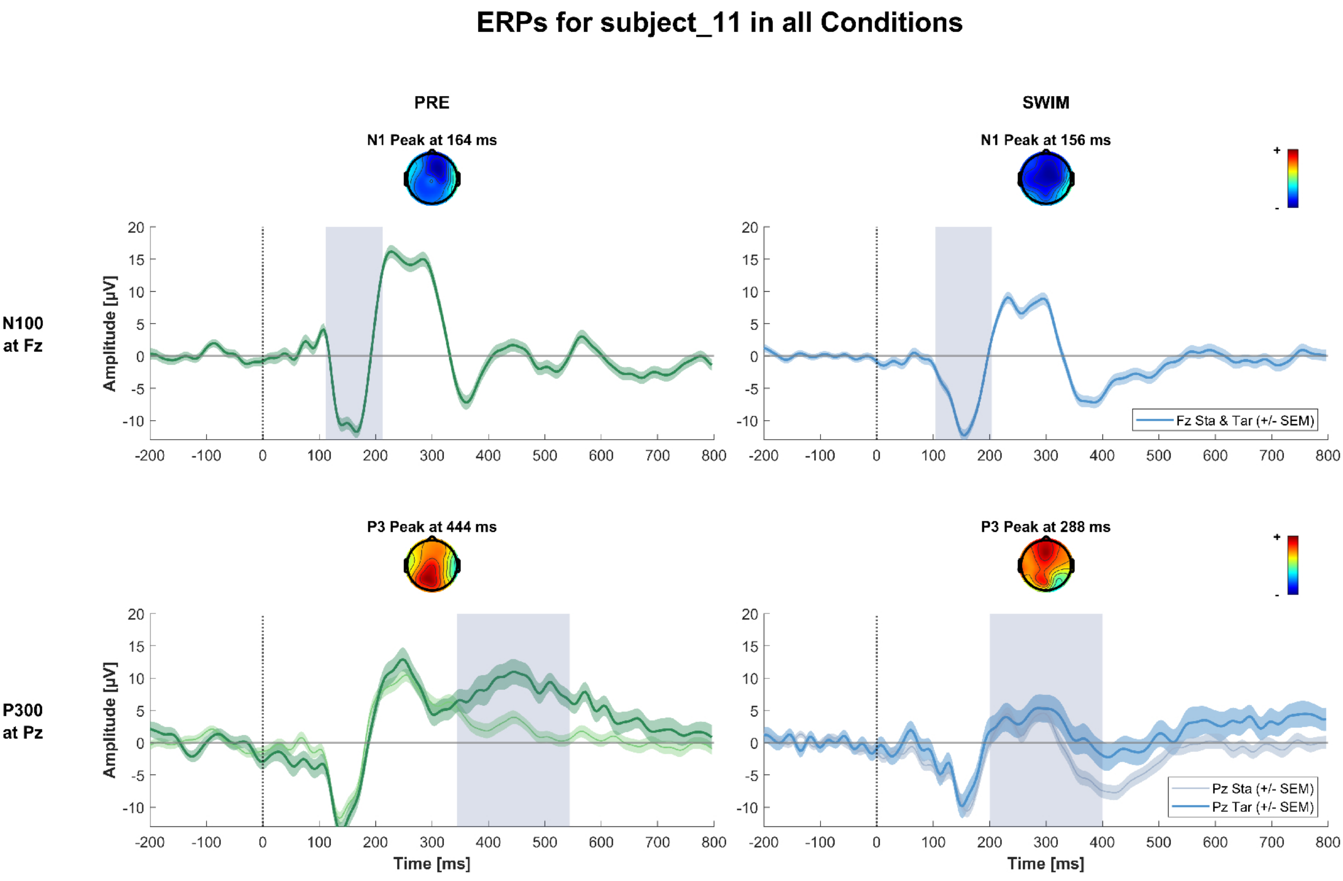

Figure S6. ERPs in every condition for Subject 11. The N100 is present in every block; the expected effect on the P300 amplitude was found only during Pre for this subject. The peak of the P300 is earlier during Swim as compared to Pre Swim.

1.3 P300 Component Statistics

	Pre		Swim		Post	
ID	Observed Difference	p-value	Observed Difference	p-value	Observed Difference	p-value
Sub 01	2.015	0.025 *	0.884	0.228	-0.029	0.52
Sub 02	0.981	0.078	0.126	0.449	-0.746	0.778
Sub 03	2.73	<0.001 ***	1.481	0.01 *	2.765	<0.001 ***
Sub 04	4.038	<0.001 ***	0.353	0.094	2.54	0.002 **
Sub 06	0.375	0.277	-0.39	0.603	1.217	0.048 *
Sub 07	5.143	<0.001 ***	3.393	0.019 *	5.076	<0.001 ***
Sub 09	1.769	<0.001 ***	0.806	0.224	1.352	0.001 **
Sub 10	-	-	0.954	0.196	0.528	0.16
Sub 11	6.104	<0.001 ***	1.91	1.96	-	-

Table S2. Subject-wise summary of p-values for the main effect of stimulus type on the ERP. In Pre Swim, 6 out of 8 subjects show the expected oddball effect. In Swim, 2 out of 9 subjects show the effect, while in Post Swim, in 5 out of 8 subjects the P300 amplitude is significantly higher after target tones as compared to standard tones.

	Interaction Amplitude (Sit - Swim)		Interaction Latency (Swim - Sit)	
ID	Observed Difference	p-value	Observed Difference	p-value
Sub 01	1.719	0.122	-46.388	1
Sub 02	-2.059	0.924	16.144	0.07
Sub 03	1.064	0.072	103.9	<0.001 ***
Sub 04	2.842	0.043 *	-21.363	0.992
Sub 06	-0.969	0.719	98.621	<0.001 ***
Sub 07	1.723	0.174	26.668	0.003 **
Sub 09	1.1	0.179	134.234	<0.001 ***
Sub 10	-0.492	0.59	65.082	<0.001 ***
Sub 11	4.618	0.105	-147.661	1

Table S3. Subject-wise summary of p-values for the interaction effect of stimulus type and motor condition on the ERP. One subject showed the hypothesized effect that the difference in the P300 amplitude between standard and target tones would be larger while participants are sitting as compared to while participants are swimming. Five out of nine subjects showed the expected effect that the latency of the P300 after target tones would be elongated during swimming as compared to sitting.

1.4 Frequency Analyses around Turns

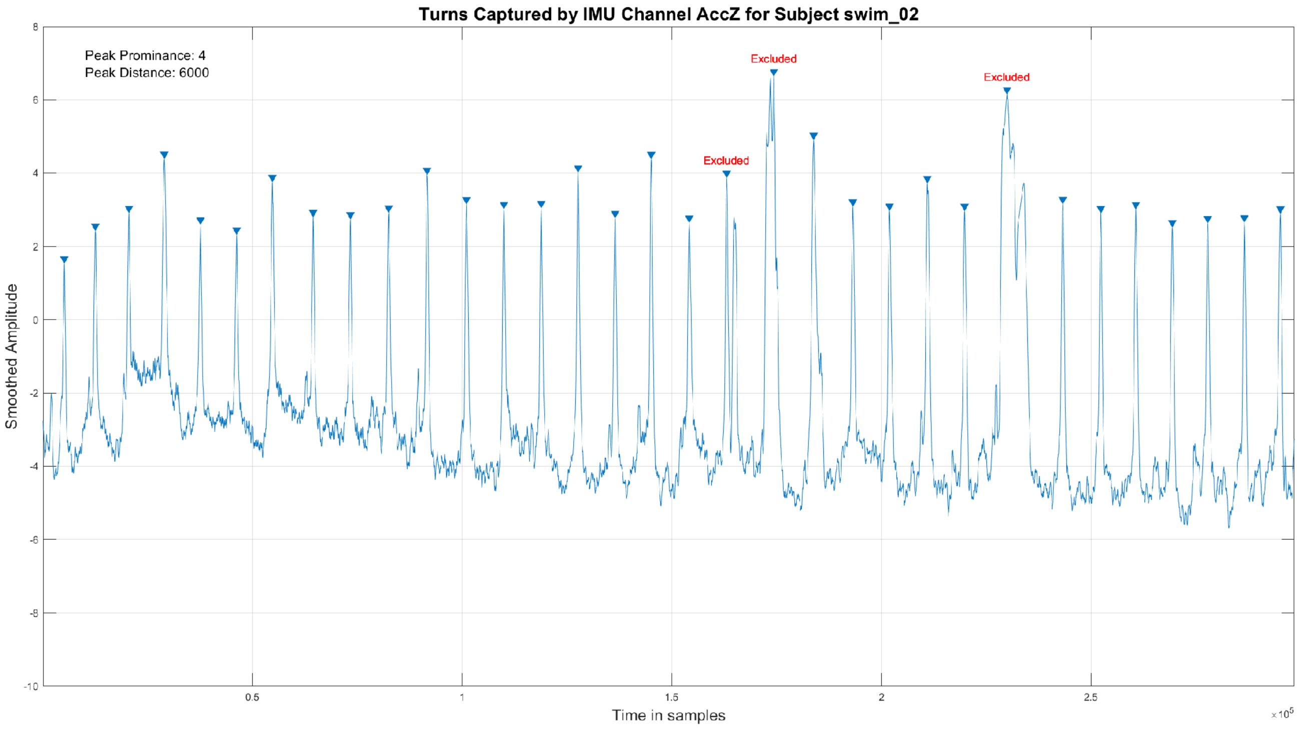

Figure S7. Example of how the markers for the turns during swimming were derived. The signal from z-axis channel of the IMU integrated in the amplifier was used to find peaks that mark a turn during swimming. Ambiguous peaks were excluded.

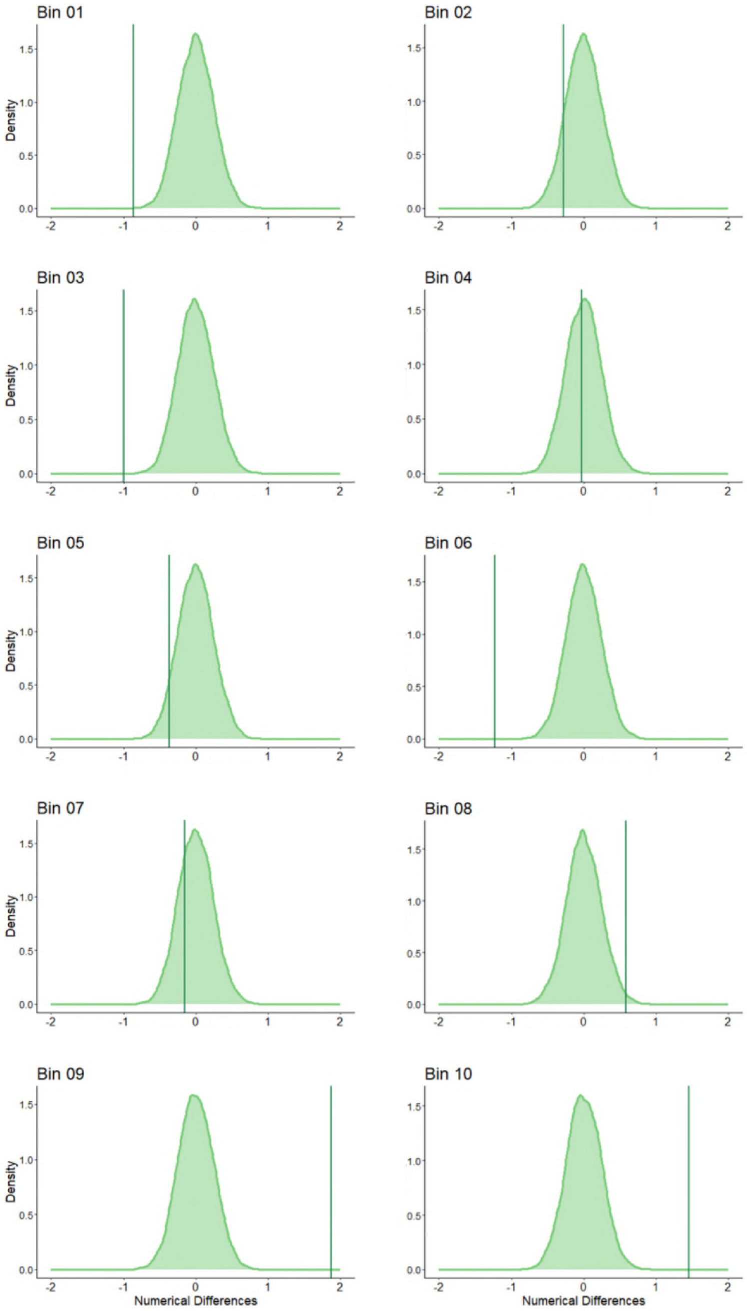

Figure S8. Result of the permutation tests for the alpha frequency band. Each subplot represents a 500 ms bin from -2500ms to +2500 ms around turns during swimming. Mean alpha power in the respective bins was compared to the mean alpha power during the whole 5s-epoch using permutation tests. The vertical line in the plots marks the observed difference between the respective bins and the whole epoch. In bins 1, 3, and 6 the alpha power is significantly lower as compared to the whole epoch. In bins 8, 9, and 10 the alpha power is significantly higher as compared to the whole epoch.

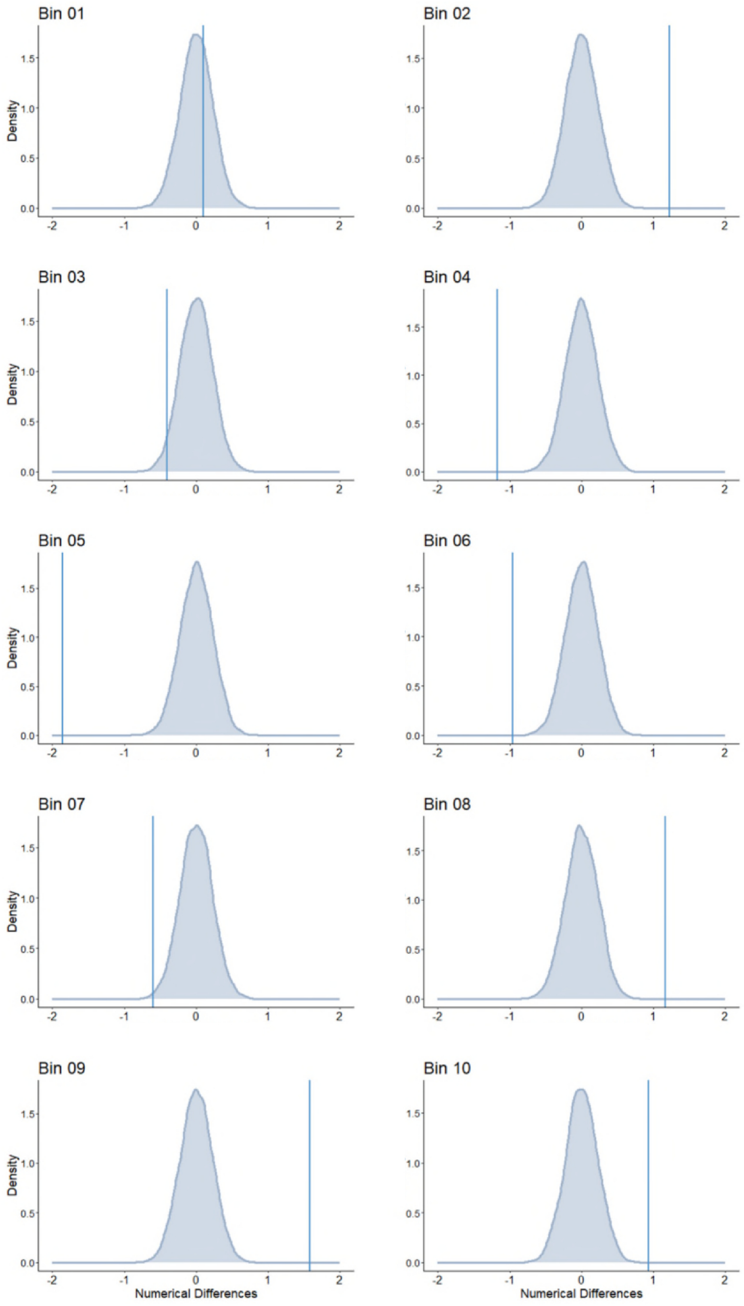

Figure S9. Result of the permutation tests for the beta frequency band. Each subplot represents a 500 ms bin from -2500ms to +2500 ms around turns during swimming. Mean beta power in the respective bins was compared to the mean beta power during the whole 5s-epoch using permutation tests. The vertical line in the plots marks the observed difference between the respective bins and the whole epoch. In bins 4,5,6, and 7 the beta power is significantly lower as compared to the whole epoch. In bins 2, 8, 9, and 10 the beta power is significantly higher as compared to the whole epoch.

	Alpha Band (Overall Mean - Bin)		Beta Band (Overall Mean - Bin)	
ID	Observed Difference	*p*-value	Observed Difference	*p*-value
Bin 01	-0.858	<0.001 ***	0.094	0.681
Bin 02	-0.276	0.254	1.222	<0.001 ***
Bin 03	-0.994	<0.001 ***	-0.410	0.069
Bin 04	-0.032	0.905	-1.169	<0.001 ***
Bin 05	-0.364	0.136	-1.853	<0.001 ***
Bin 06	-1.228	<0.001 ***	-0.953	<0.001 ***
Bin 07	-0.157	0.516	-0.603	0.008
Bin 08	0.582	0.017	1.164	<0.001 ***
Bin 09	1.874	<0.001 ***	1.577	<0.001 ***
Bin 10	1.455	<0.001 ***	0.931	<0.001 ***

Table S4. Bin-wise summary of observed differences and p-values to explore potential modulations in the alpha and beta frequency bands during turn phases. The alpha level was set to 0.0025 in line with the Bonferroni correction.
